# Antimicrobial potential of different bee product-loaded carboxymethyl chitosan nanoparticles against multidrug-resistant clinical pathogens: a comparative in vitro study

**DOI:** 10.1186/s12896-026-01159-5

**Published:** 2026-05-29

**Authors:** Asmaa K. Helmy, Asmaa S. El-Houssiny, Ahmed G. Hegazi, Nagwa M. Sidkey

**Affiliations:** 1 Botany and Microbiology Department, Faculty of Science, Al-Azhar University (Girls Branch), Cairo, Egypt; 2 Microwave Physics and Dielectrics Department, National Research Centre, Dokki, Giza Egypt; 3 Department of Zoonotic Diseases, National Research Centre, Dokki, Giza Egypt

**Keywords:** MDR, Propolis, EEP, Honey, Royal jelly, Encapsulation, MIC, CMC NPs, CMC-P NPs, CMC-H NPs, CMC-RJ NPs

## Abstract

**Background:**

Antimicrobial resistance is one of the major public health threats facing humanity. Hence, the current investigation aimed to develop alternative therapeutic strategies by integrating apitherapy with biopolymer-based nanotechnology to combat multidrug-resistant (MDR) microbial pathogens.

**Methods:**

Out of 100 clinical microbial isolates, 29 highly MDR clinical pathogens from different infections, including Gram-negative and Gram-positive bacteria, as well as fluconazole-resistant *Candida* spp., were selected for the study. Three bee products (Turkish propolis extract (P), Egyptian honey (H), and Egyptian royal jelly (RJ)) were used, and their polyphenolic contents were determined via HPLC analysis. These bee products were encapsulated within carboxymethyl chitosan nanoparticles (CMC NPs). The resulting nano-formulations, namely Carboxymethyl chitosan–Propolis extract nanoparticles (CMC-P NPs), Carboxymethyl chitosan–Honey nanoparticles (CMC-H NPs), and Carboxymethyl chitosan–Royal Jelly nanoparticles (CMC-RJ NPs), were characterized in terms of particle size, surface charge, and chemical composition. Their antimicrobial activity and preliminary cytocompatibility were evaluated using standard microbiological assays and the MTT assay, respectively.

**Results:**

HPLC analysis confirmed the presence of diverse polyphenolic compounds in the bee products. The highest concentrations were detected in the ethanolic extract of propolis (EEP) (51.0–40993 µg/g), followed by honey (1.06–1591.16 µg/g) and royal jelly (0.53–2796.37 µg/g). Among the nano-formulations, CMC-P NPs emerged as the most potent formulation (size: 100.7 nm, zeta potential: -70 mV), showing significant bactericidal activity (*P* < 0.05) against all MDR isolates. Notably, CMC-P NPs exhibited inhibition zones (17.67–31.33 mm) and MIC values (0.019–1.25 mg/mL) that were superior to standard antibiotic controls. CMC-H NPs and CMC-RJ NPs exhibited variable antimicrobial effects, depending on the pathogen and the encapsulated bee product. Indeed, all three nano-formulations demonstrated strong antimicrobial action against highly resistant *Klebsiella pneumoniae* and *Klebsiella ozaenae.* Further, MTT assay results confirmed the preliminary biocompatibility of the nanostructures, showing no significant toxicity toward RPE1 cells.

**Conclusion:**

Encapsulation of bee products within CMC NPs significantly enhances their antimicrobial performance against a wide range of MDR clinical pathogens. The developed nano-formulations exhibited suitable physicochemical properties, potent antimicrobial activity, and promising preliminary biocompatibility, supporting their potential as safe and effective antimicrobial candidates for further preclinical investigation.

**Supplementary Information:**

The online version contains supplementary material available at 10.1186/s12896-026-01159-5.

## Introduction

Antimicrobial resistance (AMR) is considered one of the most critical global public health challenges, causing over 700,000 deaths every year around the world, and is expected to cause the death of 10 million lives by 2050 [1]. The emergence of AMR has largely been driven by the inappropriate use of essential antibiotics in humans, animals, and agriculture, which promotes genetic adaptations in microbial populations and makes pathogenic infections increasingly difficult to treat. Additionally, reports highlight inadequate surveillance and monitoring of antibiotic consumption, particularly in low- and middle-income countries, as a significant contributing factor to this growing global health challenge [2]. Over recent years, extensive exposure to antimicrobials has led to the emergence and rapid spread of MDR pathogens, which have developed resistance mechanisms against several major antibiotic classes, including β-lactams, sulfamethoxazole/trimethoprim, nitrofurantoin, carbapenems, and fluoroquinolones. This resistance emerges through genetic changes in bacteria, either via mutations or through the acquisition of new resistance genes from other microbes [3, 4]. As a result, available therapeutic options to treat infections caused by these pathogens have become increasingly limited. This has increased the urgent demand for alternative approaches beyond conventional antibiotics, such as apitherapy and biopolymer-based nanotechnology, which offer innovative strategies for targeted drug delivery against MDR microbes [5, 6].

Nanotechnology-based delivery systems have occurred through the use of nanomaterials typically in ranging size of 10–1000 nm. Natural polymers such as chitosan, alginate, along with their derivatives are particularly attractive due to their biodegradability and biocompatibility [7, 8]. Polymeric nanoparticle can incorporate active compounds either through entrapment within the polymeric matrix or via surface adsorption onto the nanoparticle core [9]. This structural versatility enables improved drug solubility and stability, facilitates targeted delivery, and allows for controlled release profiles compared with conventional dosage forms [9, 10]. Carboxymethyl chitosan (CMC), a modified form of chitosan, combines the benefits of the polymer chitosan with enhanced water solubility, mucoadhesive properties, and antimicrobial activity [11]. Accordingly, carboxymethyl chitosan nanoparticles (CMC NPs) have emerged as promising nanocarriers owing to their excellent biocompatibility, biodegradability, and structural flexibility, making them suitable for a wide range of biomedical applications [12]. These nanoparticles have been widely used in vaccine and drug delivery systems, as well as antimicrobial applications, tissue engineering, and cancer therapy [8, 13, 14]. Recent studies highlight that chitosan nanoparticles can act as efficient nano-carriers for bioactive bee products, enhancing their ability to penetrate microbial biofilms and potentially improving their antimicrobial effectiveness against MDR pathogens [15–17].

Propolis, honey, and royal jelly are vital bee products, widely used in folk and modern medicine due to their antimicrobial, anti-inflammatory, antioxidant, anticancer, and immunomodulatory activities [18, 19]. Propolis (Bee’s glue) is a natural sticky bee product composed of hydrophobic resins, gums, and a complex mixture of various plant secondary metabolites such as terpenoids, alkaloids, tannins, and flavonoids, which contribute to its diverse biological activities. Bees utilize propolis as a construction material and also as a hive defensive substance against microbial invasion [18, 20]. Honey is a natural product produced from floral nectar and is primarily composed of sugars and water, along with enzymes, proteins, polyphenols, and amino acids. It is widely recognized for its nutritional value and therapeutic potential [21, 22]. Royal jelly “bee milk” is a unique secretion used to feed both young bee larvae and the queen bee throughout her life [23]. It is a complex and highly nutritious bee product, rich in proteins, carbohydrates, amino acids, and vitamins responsible for its potent biological activities [24]. Indeed, bee products are rich in polyphenolic compounds that significantly contribute to their antioxidant capacity and antimicrobial activity against a wide range of pathogenic bacteria and fungi [25].

Accordingly, the current study is a trial to integrate the therapeutic potential of multiple bee products with CMC NPs by encapsulating propolis extract, honey, and royal jelly within a unified nano-delivery platform. Unlike previous studies that primarily focused on nano-encapsulation of individual bee products, this work adopts a comparative nanoformulation approach to evaluate their relative antimicrobial performance against MDR clinical isolates from diverse infection sources.

This unified framework enables a more rational understanding of how different natural bioactive matrices behave within the same nano-system, offering insight into their comparative therapeutic potential rather than evaluating each product in isolation. Such an approach may support the development of natural adjunct strategies to combat antimicrobial resistance; however, this represents a preliminary step that warrants further optimization and investigation.

## Materials and methods

### Preparation of bee product samples (propolis extract, honey, and royal jelly)

The Egyptian honey sample was obtained from Badr apiary in Helwan, Egypt, and stored in a sterile glass container at room temperature. Turkish propolis (*Ganibal Ham Propolis*) and the Egyptian royal jelly sample were obtained from a commercial bee products market and stored in a sterile, dark glass container in the refrigerator and freezer, respectively.

The Turkish propolis sample was extracted using 70% ethanol (1:10, w/v) (Roth, Germany) at 37 °C for 7 days in the absence of light, according to Helmy et al. [26], and the resulting extract was referred to as ethanolic extract of propolis (EEP). The extraction yield was evaluated by comparing the dry weight of EEP with the initial weight of raw propolis used in percentage.

Honey and royal jelly samples were aseptically prepared at different concentrations (w/v) using sterile distilled water. Each honey or royal jelly solution was stirred by vortex to obtain a homogeneous solution.

### HPLC analysis of bee products

Qualitative determination of polyphenols of bee products (Turkish EEP, Egyptian honey, and royal jelly) was carried out using an HPLC instrument equipped with an Agilent 1260 series. The separation step was carried out using an Eclipse C18 column (4.60 mm x 250 mm) 5-micron. The mobile phase consisted of water (A) and 0.05% trifluoroacetic acid in acetonitrile (B) at a flow rate of 0.9 mL/min. The mobile phase was programmed consecutively in a linear gradient as follows: 0 min (82% A); 0–5 min (80% A); 5–8 min (60% A); 8–12 min (60% A); 12–15 min (82% A); 15–16 min (82% A); and 16–20 (82% A). The multi-wavelength detector was monitored at 280 nm. The injection volume was 5 µl for each of the sample solutions. The column temperature was maintained at 40 °C. HPLC analysis was carried out in the Central Laboratories Network, National Research Centre, Cairo, Egypt [27].

### Preparation of CMC NPs and honeybee products encapsulated within CMC NPs

According to Nguyen et al. [7], CMC NPs were synthesized using the ionic gelation method with the addition of calcium chloride (CaCl_2_) (Roth, Germany) as a cross-linker. CMC (0.5% w/v) (Santa Cruz Biotechnology, USA) was dissolved in distilled water and stirred magnetically for 1 h at room temperature. Each bee product (0.5% w/v) was then added separately to the CMC solution and stirred for 24 h at room temperature. Afterwards, CaCl_2_ (5% w/v) was added dropwise at a 1:0.8 CMC: CaCl_2_ weight ratio to induce ionic gelation, followed by stirring for 1.5 h at room temperature. The resulting nanoparticles (CMC-P NPs, CMC-H NPs, and CMC-RJ NPs) were collected, lyophilized, and stored in airtight containers at room temperature for further characterization.

### Characterization techniques for bee products encapsulated within CMC NPs

#### Particle size analysis and zeta potential measurements

The average particle diameter, size distribution, and surface charge (zeta potential) of EEP, honey, and royal jelly, as well as their corresponding nano-formulations (CMC-P NPs, CMC-H NPs, and CMC-RJ NPs) were evaluated using a Nano-ZS particle size analyzer (Malvern Instruments Ltd., UK). Each sample was prepared in distilled water and ultrasonicated for 3 min to to obtain homogeneous dispersions prior to analysis. Measurements were performed on aqueous suspensions at 25 °C. All determinations were carried out in triplicate, and the results were expressed as mean values.

#### Attenuated total reflection-fourier transform infrared (ATR-FTIR) spectroscopy

ATR–FTIR spectroscopy was employed to investigate the interactions between the bee products (EEP, honey, and royal jelly) and CMC nanoparticles. Spectra of the bee products, along with CMC NPs and their respective nano-formulations (CMC-P NPs, CMC-H NPs, and CMC-RJ NPs), were recorded using a Bruker VERTEX 80 ATR–FTIR spectrophotometer (Germany) equipped with a Platinum Diamond ATR crystal as the internal reflector. Samples were carefully applied to the ATR crystal, and spectra were collected over a scanning range of 400–4000 cm⁻¹ at a resolution of 4 cm⁻¹.

### Antimicrobial assay for bee products encapsulated within CMC NPs

#### The agar-well diffusion method

Twenty-nine extreme MDR clinical microbial isolates were selected from 100 random clinical isolates from a previous study of ours [3] as follows: 19 Gram-negative bacteria (1 *Acinetobacter lwoffii* (I_9_ Al_1_*), 3 *Acinetobacter baumannii* (I_73_ Ab_2_*, I_87_ Ab_3_*, and I_88_ Ab_4_*), 3 *E. coli* (I_12_ E_6_, I_14_ E_8_, and I_93_E_34_), 1 *Hafnia alvei* (I_65_ H_1_), 4 *Klebsiella ozaenae* (I_2_ Ko_1_*, I_80_ Ko_2_, I_83_ Ko_3_, and I_85_ Ko_4_), 3 *Klebsiella pneumoniae* (I_33_ Kp_4_, I_56_ Kp_6_*, and I_89_Kp_8_), 2 *Pseudomonas aeruginosa* (I_37_ P_3_* and I_100_ P_8_), 1 *Serratia liquefaciens* (I_38_ Sel_1_*), and 1 *Serratia rubidaea* (I_48_ Ser_1_*)); 4 Gram-positive bacteria (2 coagulase negative (CON) *Staphylococcus (*I_84_ St_− 5_ and I_92_ St_− 6_*)* and *Staphylococcus aureus* (I_41_ St_2_* and I_44_ St_3_)); and eventually 6 yeast isolates (3 *Candida albicans* (I_39_ Ca_7_, I_60_ Ca_8_*, and I_72_ Ca_12_*), 1 *Candida glabrata* (I_66_ Cg_1_*), 1 *Candida krusei* (I_59_ Ck_1_), and 1 *Candida tropicalis* (I_23_ Ct_1_*)). Moreover, microbial isolates obtained from COVID-19 patients were mentioned with a star (*), and the antimicrobial resistance profiles of the 29 MDR pathogens are shown in Supplementary Table S1.

The antimicrobial activity of CMC NPs (10 mg/mL), free bee products (10 mg/mL), and bee products-loaded CMC NPs (10 mg/mL) was determined by the agar-well diffusion method on Mueller-Hinton agar against 29 MDR clinical microbial isolates, according to NCCLS [28] guidelines. The concentrations are expressed as the total mass concentration of the tested samples, including nano-formulations. In addition, amoxicillin-clavulanic acid (AMC 30), ciprofloxacin (CIP 5), and fluconazole 25 µg discs were used as positive controls, the zone of inhibition was measured, and results were interpreted according to the Clinical and Laboratory Standards Institute [29]. Each clinical microbial strain was freshly prepared at a concentration of 1.5 × 10^8^ CFU/mL and swabbed over the surface of Mueller-Hinton agar plates. Wells were made with a diameter of 8 mm and a volume of 100 µL of Turkish EEP, Egyptian honey, Egyptian royal jelly, CMC NPs, CMC-P NPs, CMC-H NPs, and CMC-RJ NPs were dropped in each plate well. The plates were kept in the refrigerator for 1–2 h before incubation to allow the diffusion of the tested samples. Then, they were incubated at 35–37 °C for 18–24 h, and the inhibition zone diameters (IZDs) were measured in mm. The experiment was carried out in triplicate, and the results were expressed as mean ± standard error (SE).

#### MIC and MBC determination

The minimum inhibitory concentrations (MIC) and minimum bactericidal concentration (MBC) values were determined for one isolate from each bacterial and yeast species, specifically selecting the most resistant isolate. These included 9 Gram-negative bacteria (*A. lwoffii* I_9_ Al_1_^*^, *A. baumannii* I_87_ Ab_3_^*^, *E. coli* I_93_ E_33_, *H. alvei* I_65_ H_1_, *P. aeruginosa* I_37_ P_3_^*^, *K. pneumonia* I_33_ Kp_4_, *K. ozaenae* I_2_ Ko_1_^*^, *S. liquefaciens* I_38_ Sel_1_^*^, and *S. rubidaea* I_48_ Ser_1_^*^), 2 Gram-positive bacteria (*S. aureus* I_41_ St_2_^*^ and CON *Staphylococcus* I_84_ St_− 5_), and 4 *Candida* spp. (*C. albicans* I_72_ Ca_12_^*^, *C. glabrata* I_66_ Cg_1_^*^, *C. krusei* I_59_ Ck_1_, and *C. tropicalis* I_23_ Ct_1_^*^).

MIC determination of CMC NPs and bee products loaded CMC NPs was carried out using the microdilution method on 15 of the most MDR clinical microbial isolates under study. Standardized microbial suspensions (1.5 × 10⁸ CFU/mL) were incubated in 96-well microtiter plates containing broth Mueller-Hinton agar media supplemented with various concentrations (0.01-10 mg/mL) of CMC NPs, CMC-P NPs, CMC-H NPs, and CMC-RJ NPs for 24 h at 35–37 °C. The MIC values for the bee products without encapsulation had been previously determined in a previous study of ours with different concentrations of EEP (0.1–125 mg/mL), honey (10–750 mg/mL), and royal jelly (5–500 mg/mL) [26]. Wells without nanoparticles were used as negative controls. MIC values were estimated by visual and spectroscopic methods using absorbance measurements at 620 nm, the lowest concentration that suppressed microbial growth. For MBC determinations, 5 µL of the previous wells was transferred on Mueller-Hinton agar, and after overnight incubation, the lowest concentration that revealed no visible bacterial growth (99% inhibition) was regarded as MBC.

### Assessment of cell viability of bee product-loaded CMC nanoparticles using the MTT assay

The effect of bee products encapsulated within CMC NPs on the viability of normal human cells was evaluated by colorimetric MTT (3-[4,5-dimethylthiazol-2]-2,5- diphenyltetrazolium bromide) assay, according to **Mosmann et al.** [30] and **Buranaamnuay** [31]. In the cell culture experiment, human normal retinal pigment epithelial (RPE1) cells (Karolinska Centre, Department of Oncology and Pathology, Karolinska Institute and Hospital, Stockholm) were maintained in standard conditions in DMEM-F12 medium. Then, using a water-jacketed carbon dioxide incubator, cells were seeded at a concentration of 10^4^ cells/well in 96-well plates at 37 °C under 5% CO_2_. After that, normal RPE1 cells were incubated with different concentrations (3, 6.25, 12.5, 25, 50, and 100 µg/mL) of CMC-P NPs, CMC-H NPs, and CMC-RJ NPs for 48 h. Cells in media alone without treatment were acting as a negative control. A positive control ‘doxorubicin’ was used as a known cytotoxic natural agent that gives 100% toxicity under the same conditions. After 48 h of incubation, MTT was added to each group, and the cells were incubated for 4 hours. The formazan crystals formed were then dissolved with sodium dodecyl sulfate (SDS). Using an Elisa reader (Bio-Rad Laboratories Inc., model 3350, Hercules, California, USA), the absorbance was measured at 595 nm. The experiment was carried out in triplicate and results are expressed as Mean ± SE.

### Data analysis

Data were expressed as mean (triplicate) ± standard error (SE). The distribution of the data was confirmed to be normal, and statistical significance was determined using one-way analysis of variance (ANOVA) followed by the Least Significant Difference (LSD) test. Statistical analyses were performed using CoStat software (version 6.45 for Windows). A *P* value of < 0.05 was considered statistically significant for all analysis.

## Results

### Extraction yield of EEP and HPLC analysis of bee products

**Fig. 1:**
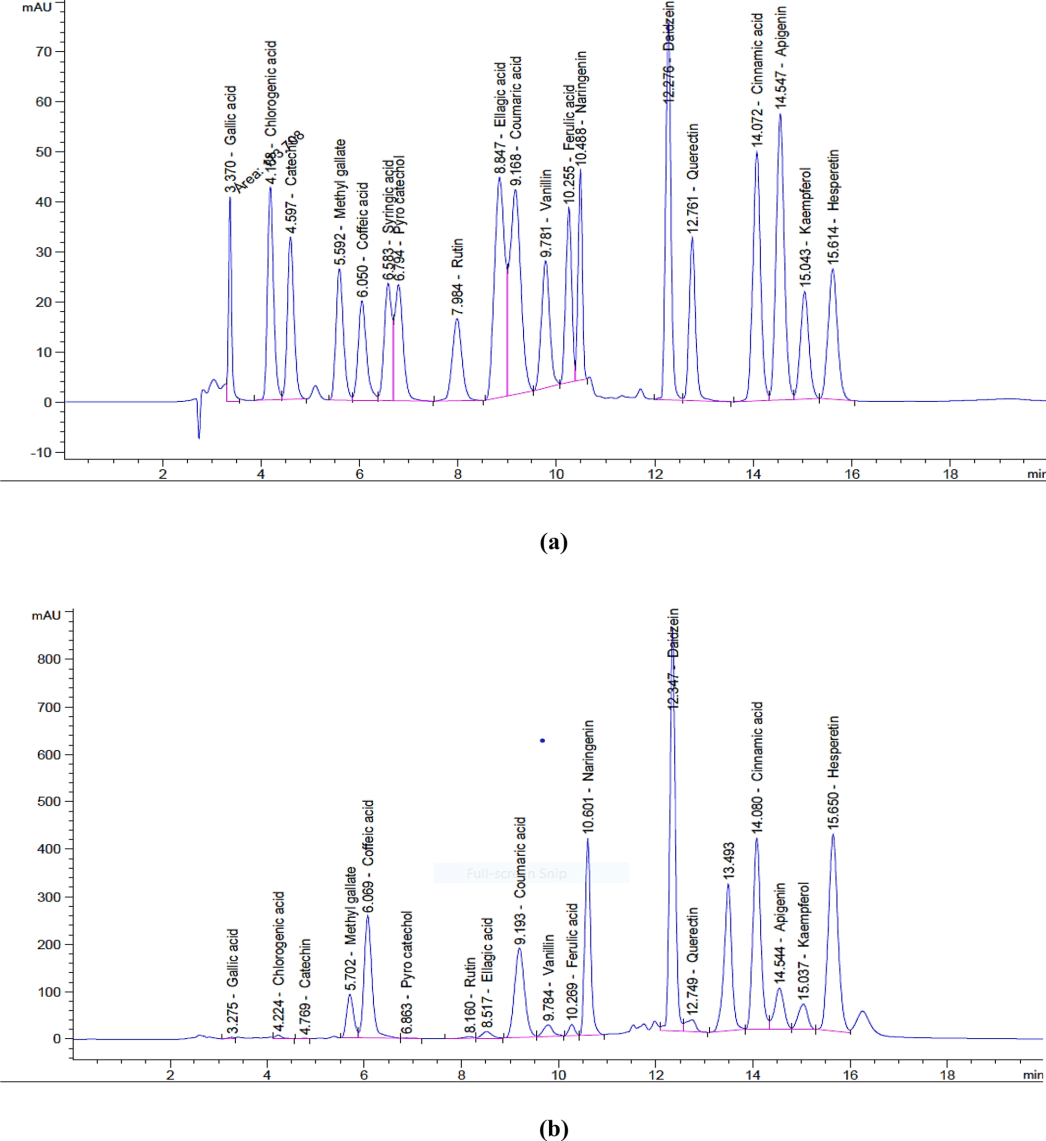

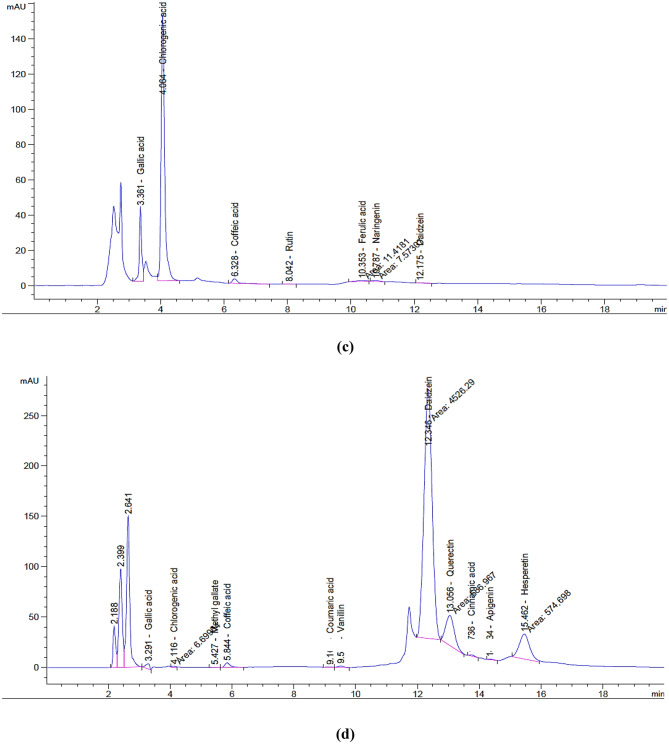
HPLC chromatogram with identified marker compounds for **(a)** reference standards polyphenol compounds, **(b)** Turkish propolis, **(c)** Egyptian honey, and **(d)** Egyptian royal jelly

Turkish propolis sample was extracted using 70% ethanol, reporting an extraction yield of 59.3%. The qualitative analysis of polyphenolic compounds in bee products (Turkish EEP, Egyptian honey, and Egyptian royal jelly) was performed using HPLC analysis. The HPLC chromatogram of Turkish EEP (Table 1; Fig. 1-b) revealed the presence of 11 phenolic acids and 7 flavonoid compounds in the range of 51.0–40.993 µg/g. Daidzein (40993 µg/g) and naringenin (40707 µg/g) were the most abundant constituents in Turkish EEP, followed by hesperetin (32955 µg/g), caffeic acid (22369 µg/g), apigenin (8798 µg/g), coumaric acid (8478 µg/g), cinnamic acid (8254 µg/g), kaempferol (5538 µg/g), methyl gallate (5047 µg/g), querctin (4967 µg/g), and ellagic acid (3581 µg/g). In comparison, pyro catechol (75.6 µg/g) and catechin (51.0 µg/g) were present in minor amounts.

In the case of Egyptian honey (Table 1; Fig. 1-c), the chromatogram showed the presence of 3 flavonoid compounds and 4 phenolic acids, with concentrations ranging from 1.06 to 1591.16 µg/g. Indeed, Egyptian honey contains a high content of chlorogenic acid (1591.16 µg/g) and lower amounts of gallic acid (185.67 µg/g), caffeic acid (24.89 µg/g), naringenin (9.13 µg/g), ferulic acid (7.80 µg/g), rutin (1.87 µg/g), and daidzein (1.06 µg/g).

Meanwhile, the Egyptian royal jelly (Table 1; Fig. 1-d) exhibited a polyphenolic profile of 7 phenolic acids and 4 flavonoid compounds in the range of 0.53–2796.37 µg/g. Daidzein (2796.37 µg/g) and querctin (808.48 µg/g) were the most predominant constituents in royal jelly, followed by hesperetin (335.01 µg/g), caffeic acid (40.15 µg/g), and gallic acid (38.64 µg/g). In comparison, chlorogenic acid (9.17 µg/g), vanillin (6.90 µg/g), apigenin (3.99 µg/g), cinnamic acid (2.51 µg/g), coumaric acid (1.07 µg/g), and methyl gallate (0.53 µg/g) were present in minor amounts.

**Table 1:** HPLC analysis results of Turkish EEP, Egyptian honey, and Egyptian royal jelly

Identified compounds	Retention time (min)				Concentration		
	Standard	EEP	Honey	Royal Jelly	EEP (µg/g)	Honey (µg/g)	Royal Jelly (µg/g)
Gallic acid	3.370	3.275	3.361	3.291	224	185.67	38.64
Chlorogenic	4.188	4.224	4.064	4.116	908	**1591.16**	9.17
Catechin	4.597	4.769	ND	ND	51	ND	ND
Methyl gallate	5.592	5.702	ND	5.427	5.047 × 10^3^	ND	0.53
Caffeic acid	6.050	6.069	6.069	5.844	**22.369 × 10** ^**3**^	24.89	40.15
Pyro catechol	6.794	6.863	ND	ND	76	ND	ND
Rutin	7.984	8.160	8.042	ND	551	1.87	ND
Ellagic acid	8.847	8.517	ND	ND	3.581 × 10^3^	ND	ND
Coumaric acid	9.168	9.193	ND	9.166	**8.478 × 10** ^**3**^	ND	1.07
Vanillin	9.781	9.784	ND	9.516	1.502 × 10^3^	ND	6.90
Ferulic acid	10.255	10.269	10.353	ND	1.341 × 10^3^	7.80	ND
Naringenin	10.488	10.601	10.787	ND	**40.707 × 10** ^**3**^	9.13	ND
Daidzein	12.276	12.347	12.175	12.346	**40.993 × 10** ^**3**^	1.06	**2796.37**
Querctin	12.761	12.749	ND	13.056	4.967 × 10^3^	ND	**808.48**
Cinnamic acid	14.072	14.080	ND	13.736	**8.254 × 10** ^**3**^	ND	2.51
Apigenin	14.547	14.544	ND	14.334	**8.798 × 10** ^**3**^	ND	3.99
Kaempferol	15.043	15.037	ND	ND	5.538 × 10^3^	ND	ND
Hesperetin	15.614	15.650	ND	15.462	**32.955 × 10** ^**3**^	ND	**335.01**

ND: Not detected

### Characterization techniques for bee products encapsulated within CMC NPs

#### Particle size analysis

**Fig. 2:**
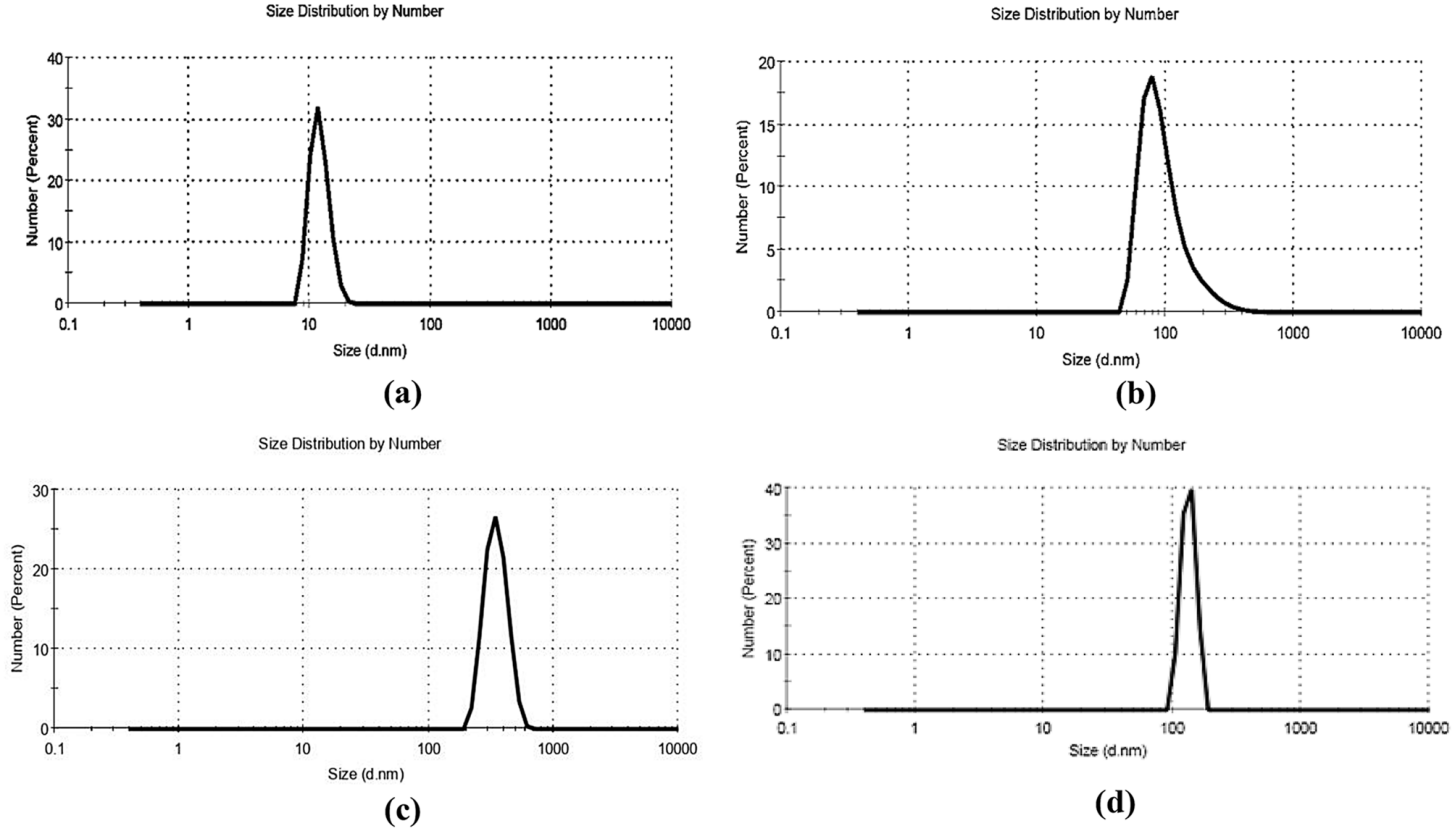
Particle size of **(a)** CMC NPs, **(b)** CMC-P NPs, **(c)** CMC-H NPs, and **(d)** CMC-RJ NPs

The particle size and size distribution of CMC NPs, CMC-P NPs, CMC-H NPs, and CMC-RJ NPs were determined by dynamic light scattering (DLS) measurements. According to Fig. 2 (a, b, c, and d), it was found that CMC NPs, CMC-P NPs, CMC-RJ NPs, and CMC-H NPs have an average diameter of 12.16, 100.7, 134.5, and 350.8 nm, respectively. Also, the Polydispersity index (PDI) values for CMC NPs, CMC-P NPs, CMC-H NPs, and CMC-RJ NPs were 0.655, 0.282, 0.51, and 0.528, respectively.

#### Zeta potential measurement

**Fig. 3:**
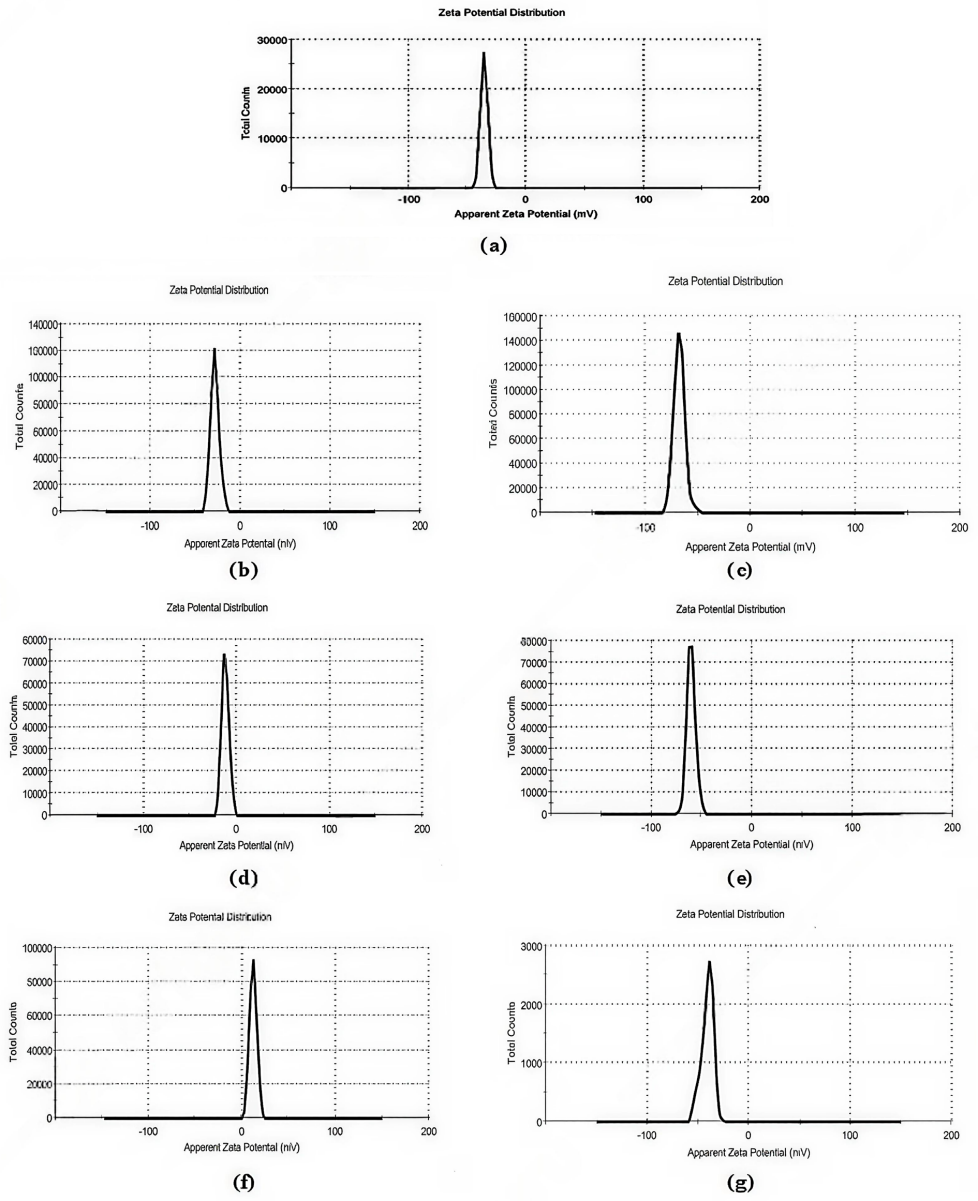
Zeta potential of **(a)** CMC NPs − 35.8, **(b)** EEP − 29 mV, **(c)** CMC-P NPs − 67 Mv, **(d)** Honey − 11 mV, **(e)** CMC-H NPs − 65 mV, **(f)** Royal Jelly + 12 mV, and **(g)** CMC-RJ NPs − 45 mV

The surface charge of EEP, honey, royal jelly, CMC NPs, and their respective loaded nanoparticles (CMC-P NPs, CMC-H NPs, and CMC-RJ NPs) was determined by zeta potential analysis. According to Fig. 3, the CMC NPs (Fig. 3-a) exhibited a zeta potential of − 35.8 ± 2.98 mV. Moreover, Turkish EEP and Egyptian honey (Figs. 3-b and 3-d) showed negative zeta potentials of -28.2 ± 4.78 mV and − 11.9 ± 3.85 mV, respectively, while Egyptian royal jelly (Fig. 3-f) demonstrated a slightly positive zeta potential of 12.0 ± 3.54 mV. After the encapsulation of EEP, honey, and royal jelly within CMC NPs, the zeta potential values increased to -67 ± 7.0, − 65.5 ± 4.23, and − 45.0 ± 5.81 mV, respectively; Fig. 3 (c, e, and g).

The significant increase in the negative zeta potential values for all loaded nano-formulations (ranging from − 45.0 to -67.0 mV) compared to CMC NPs alone indicates an enhancement in the colloidal stability of the particles after encapsulation.

#### Attenuated total reflection–fourier transform infrared (ATR–FTIR) spectroscopy

**Fig. 4:**
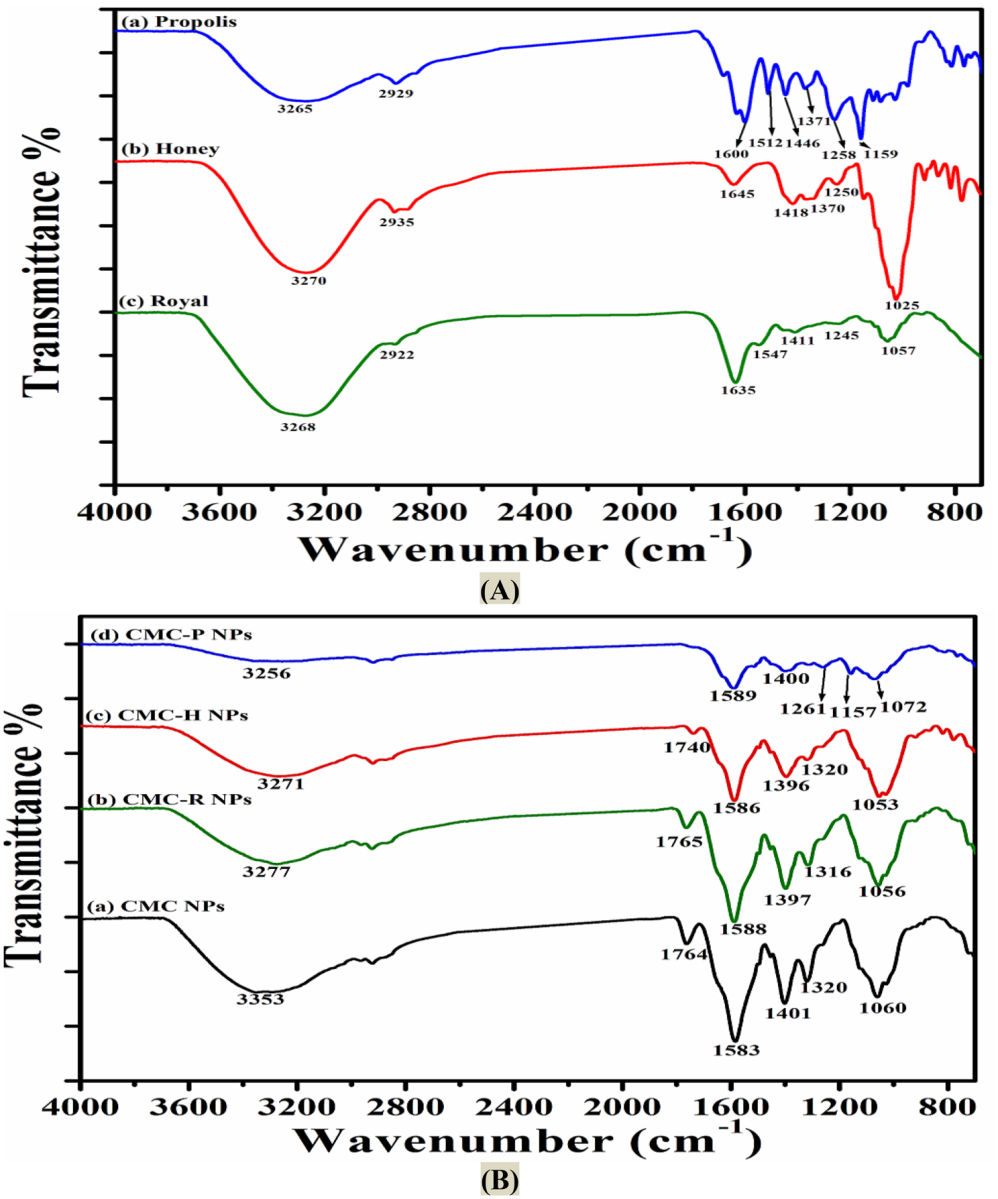
FTIR of **(A)** bee products (EEP, honey, and royal jelly) and **(B)** CMC-P NPs, CMC-H NPs, CMC-RJ NPs, and CMC NPs

The FTIR spectra of EEP, honey, royal jelly, CMC NPs, and their respective loaded nanoparticles (CMC-P NPs, CMC-H NPs, and CMC-RJ NPs) are shown in Fig. 4. In Fig. 4-A, the FTIR spectrum of EEP illustrated a peak at 3265 cm^− 1^ assigned to O-H stretching vibrations of phenolic compounds and carbohydrates [32]. The peaks at 2929 cm^− 1^ and 2858 cm^− 1^ were attributed to the stretching vibration of methyl (CH_3_) and methylene (CH_2_) groups of lipids and fatty acids. The peaks at 1681, 1631, 1600, and 1512 cm^−1^were due to C = O of carbohydrates and C = C stretching vibration, which were present in large amounts in aromatic compounds such as flavonoids [33]. On the other hand, there were two distinct peaks at 1446 cm^− 1^ and 1371 cm^− 1^, which attributed to the asymmetrical and symmetrical bending vibration of CH_3_. The peaks at 1258, 1159, 1111, and 1028 cm^− 1^ were due to the C-O stretching vibration of phenols, secondary alcohol, and primary alcohol [34].

Figure 4-A also showed the FTIR spectrum of honey with bands associated with the chemical groups of components present in the honey, such as amino acids, carbohydrates, and simple carboxylic acids. The band observed at 3270 cm^− 1^ was due to the stretching of O-H groups in water molecules. The band at 2935 cm^− 1^ was responsible for the C-H stretching of carboxylic acids. The band at 1645 cm^− 1^ corresponded to O-H stretching/bending in water [35], ketone (C = O) stretching in fructose, and aldehyde (CH = O) stretching vibrations in glucose. The peaks at 1249 cm^− 1^ and 1025 cm^− 1^ were due to the stretching vibration of C–C and C–O, respectively, in the carbohydrate fraction of the honey [36].

Further, regarding the spectrum of royal jelly (Fig. 4-A), the band observed at 3268 cm^− 1^ resulted from O-H groups of water and N-H stretching vibrations of amines. The strong absorption band at 1635 cm^− 1^ corresponded to C = O stretching vibrations of Amide I arising from the backbone conformation of proteins. The peak at 1547 cm^− 1^ resulted from N-H bending and C-N stretching vibrations of Amide II, which can be used as a key spectral feature for the quantification of royal jelly [37]. In addition, the peak at 1057 cm^− 1^ related to the RCO-OH groups of carboxylic acids in 10-HDA, benzoic acid, and gluconic acid [38].

In comparison, Fig. 4-B showed the characteristic peaks of CMC NPs at 3353 cm^− 1^ due to the stretching vibration of the NH_2_ and O-H groups. The peak at 1764 cm^− 1^ corresponded to the stretching vibration of carbonyl groups. The strong peaks at 1583 cm^− 1^ and 1401 cm^− 1^ corresponded to the N-H bending and stretching vibration of COO-, respectively. The peaks at 1320 cm^− 1^ and 1060 cm^− 1^ were attributed to the C-O stretching vibration in primary alcohols [13, 39].

In the spectrum of CMC-RJ NPs, CMC-H NPs, and CMC-P NPs; Fig. 4-B, the characteristic bands of CMC NPs weren’t affected by the entrapment of bee products, as they were slightly shifted from (1583 and 1401 cm^− 1^) to (1589 and 1400 cm^− 1)^ in CMC-P NPs, to (1586 and 1396 cm^− 1^) in CMC-H NPs, and to (1588 and 1397 cm^− 1^) in CMC-RJ NPs. Hence, no new chemical bond was formed between the functional groups in the bee products and the polymer after preparing the nanoparticles. By comparing the FTIR spectra of CMC NPs (Fig. 4-A) and CMC-P NPs (Fig. 4-B), the disappearance of peaks at 1764 and 1320 cm^− 1^ in the CMC-P NPs spectrum was observed, corresponding to the stretching vibrations of C = O and C-O groups, respectively. Further, new peaks appeared at 1261 and 1157 cm^− 1^ in the CMC-P NPs spectrum assigned to the C-O stretching vibration of phenols and secondary alcohols (characteristic peaks within the EEP spectrum), indicating the successful incorporation of propolis. Moreover, there was a noticeable reduction in the intensity of peaks at 1589, 1400, and 1072 cm^− 1^ in the CMC-P NPs spectrum and 1740, 1586, 1396, and 1320 cm^− 1^ in the CMC-H NPs spectrum, further confirming the successful encapsulation of EEP and honey within CMC NPs.

### Antimicrobial assays

#### Antimicrobial activity of EEP encapsulated within CMC NPs

##### The agar-well diffusion method

From the results presented in Table 2, Turkish EEP exhibited a good bactericidal effect at a low concentration (10 mg/mL) against all 29 MDR microbial isolates, with IZD ranging from 9.33 mm in *A. lwoffii* and *S. rubidaea* to 20.33 mm in *A.baumannii*. Furthermore, CMC NPs at the same concentration (10 mg/mL) showed significant antimicrobial activity against MDR *Klebsiella* spp. (*K. pneumonia* and *K. ozaenae*) and one isolate of *A. baumannii*, with IZD values ranging 13.67–19.33 mm. However, MDR *E. coli*,* H. alvei*,* P. aeruginosa*,* Serratia spp.*,* Staphylococcus spp.*, and *Candida* spp. were not affected by CMC NPs at this concentration. Further, Amoxicillin-clavulanic acid (AMC 30), ciprofloxacin (CIP 5), and fluconazole (25-µg) discs were used as positive controls; however, they showed no inhibitory effect on all tested MDR clinical pathogens, as IZD below 12 mm for most antibiotics, indicating resistance according to the Clinical and Laboratory Standards Institute (CLSI) breakpoints.

When EEP was encapsulated within CMC NPs according to Table 2 and supplementary Plate 1, the resulting CMC-P NPs formulation exhibited enhanced antimicrobial activity compared to pure EEP. At a concentration of 10 mg/mL, CMC-P NPs demonstrated excellent antimicrobial action on all MDR clinical microbial isolates, with IZD ranging 17.67–31.33 mm. Indeed, *A. baumannii*, *H. alvei*, *Klebsiella* spp. (*K. pneumonia* and *K. ozaenae*), and *Staphylococcus* spp. were the most sensitive with IZD values between 25.67 and 31.33 mm, followed by *Serratia* spp. (IZD = 19.33–23.00 mm), *E. coli* (IZD = 19.00–21.00 mm), and *P. aeruginosa* (IZD = 18.33–20.00 mm). Among the Candida spp., *C. krusei* was more susceptible (IZD = 28.00 mm) than other *Candida* spp. (IZD = 19.67–25.33 mm).

The p-values ​​revealed that CMC-P NPs treatment option exhibited a significantly greater antimicrobial potential than either EEP or CMC NPs alone against all tested pathogens. These statistical findings support that the encapsulation of EEP within CMC NPs enhances its antimicrobial efficacy, making the CMC-P NPs formulation a more effective therapeutic option.

**Table 2:** Antimicrobial activity of positive controls, EEP, CMC NPs, and CMC-P NPs at 10 mg/mL on MDR gram-negative, gram-positive bacteria, and yeast clinical isolates

Pathogen	Mean (mm) ± SE					
	CMC NPs	EEP	CMC-*P* NPs	AMC 30	CIP 5	Flu 25
*A. lwoffii*						
I_9_ Al_1_^*^	0.00^c^ ± 0.00	9.33^b^ ± 0.67	**18.33** ^**a**^ ** ± 0.33**	0.00 ± 0.00	0.00 ± 0.00	NA
*A. baumannii*						
I_73_ Ab_2_^*^	0.00^c^ ± 0.00	18.00^b^ ± 0.58	**31.33** ^**a**^ ** ± 1.33**	0.00 ± 0.00	0.00 ± 0.00	NA
I_87_ Ab_3_^*^	13.67^c^ ± 0.33	20.33^b^ ± 0.33	**29.67** ^**a**^ ** ± 1.20**	0.00 ± 0.00	9.33 ± 0.67	NA
I_88_ Ab_4_^*^	0.00^c^ ± 0.00	15.33^b^ ± 0.88	**25.67** ^**a**^ ** ± 0.88**	0.00 ± 0.00	0.00 ± 0.00	NA
*E. coli*						
I_12_ E_6_	0.00^c^ ± 0.00	10.67^b^ ± 0.33	**21.00** ^**a**^ ** ± 0.58**	0.00 ± 0.00	0.00 ± 0.00	NA
I_14_ E_8_	0.00^c^ ± 0.00	12.00^b^ ± 0.58	**17.67** ^**a**^ ** ± 0.33**	0.00 ± 0.00	0.00 ± 0.00	NA
I_93_ E_34_	0.00^c^ ± 0.00	12.67^b^ ± 0.88	**19.00** ^**a**^ ** ± 0.58**	0.00 ± 0.00	0.00 ± 0.00	NA
*H. alvei*						
I_65_ H_1_	0.00^c^ ± 0.00	14.33^b^ ± 0.33	**29.00** ^**a**^ ** ± 0.58**	0.00 ± 0.00	0.00 ± 0.00	NA
*K. pneumoniae*						
I_33_Kp_4_	19.33^b^ ± 0.33	18.67^b^ ± 0.33	**29.00** ^**a**^ ** ± 0.58**	7.33 ± 0.33	7.00 ± 0.58	NA
I_56_ Kp_6_^*^	18.67^b^ ± 0.88	18.00^b^ ± 0.58	**28.67** ^**a**^ ** ± 0.33**	0.00 ± 0.00	10.00 ± 0.00	NA
I_89_ Kp_8_^*^	18.00^b^ ± 0.58	18.33^b^ ± 0.33	**30.00** ^**a**^ ** ± 0.58**	0.00 ± 0.00	0.00 ± 0.00	NA
*K. ozaenae*						
I_2_ Ko_1_^*^	18.00^b^ ± 1.15	18.33^b^ ± 0.67	**28.67** ^**a**^ ** ± 0.33**	0.00 ± 0.00	0.00 ± 0.00	NA
I_80_ Ko_2_	17.33^b^ ± 0.88	17.33^b^ ± 1.20	**28.67** ^**a**^ ** ± 0.88**	0.00 ± 0.00	8.33 ± 0.33	NA
I_83_ Ko_3_	18.00^b^ ± 0.58	19.33^b^± 0.33	**28.00** ^**a**^ ** ± 0.58**	0.00 ± 0.00	7.67 ± 0.88	NA
I_85_ Ko_4_	17.67^b^ ± 0.33	17.00^b^ ± 0.58	**27.00** ^**a**^ ** ± 1.15**	0.00 ± 0.00	0.00 ± 0.00	NA
*P. aeruginosa*						
I_37_ P_3_^*^	0.00^c^ ± 0.00	13.67^b^ ± 0.33	**18.33** ^**a**^ ** ± 0.67**	0.00 ± 0.00	0.00 ± 0.00	NA
I_100_ P_8_	0.00^c^ ± 0.00	13.00^b^ ± 0.58	**20.00** ^**a**^ ** ± 1.15**	0.00 ± 0.00	0.00 ± 0.00	NA
*S. liquefaciens*						
I_38_ Sel_1_^*^	0.00^c^ ± 0.00	11.33^b^ ± 0.33	**23.00** ^**a**^ ** ± 1.00**	0.00 ± 0.00	0.00 ± 0.00	NA
*S. rubidaea*						
I_48_ Ser_1_^*^	0.00^c^ ± 0.00	9.67^b^ ± 0.33	**19.33** ^**a**^ ** ± 1.20**	0.00 ± 0.00	0.00 ± 0.00	NA
CON *Staphylococcus* spp.						
I_84_ St_− 5_	0.00^c^ ± 0.00	15.00^b^ ± 0.58	**26.67** ^**a**^ ** ± 0.33**	0.00 ± 0.00	0.00 ± 0.00	NA
I_92_ St_− 6_	0.00^c^ ± 0.00	15.67^b^ ± 0.88	**29.00** ^**a**^ ** ± 0.58**	6.00 ± 0.58	8.33 ± 1.20	NA
*S. aureus*						
I_41_ St_2_^*^	0.00^c^ ± 0.00	14.67^b^ ± 1.20	**26.33** ^**a**^ ** ± 0.88**	0.00 ± 0.00	9.67 ± 1.45	NA
I_44_ St_3_	0.00^c^ ± 0.00	13.00^b^ ± 0.58	**26.67** ^**a**^ ** ± 1.20**	0.00 ± 0.00	0.00 ± 0.00	NA
*C. albicans*						
I_39_ Ca_7_	0.00^c^ ± 0.00	10.33^b^ ± 0.33	**19.67** ^**a**^ ** ± 1.20**	NA	NA	9.33 ± 0.67
I_60_ Ca_8_^*^	0.00^c^ ± 0.00	10.00^b^ ± 0.58	**19.67** ^**a**^ ** ± 0.33**	NA	NA	7.00 ± 0.58
I_72_ Ca_12_^*^	0.00^c^ ± 0.00	12.33^b^ ± 0.33	**21.67** ^**a**^ ** ± 0.88**	NA	NA	0.00 ± 0.00
*C. glabrata*						
I_66_ Cg_1_^*^	0.00^c^ ± 0.00	11.33^b^ ± 0.67	**21.00** ^**a**^ ** ± 0.58**	NA	NA	0.00 ± 0.00
*C. krusei*						
I_59_ Ck_1_	0.00^c^ ± 0.00	14.00^b^ ± 0.58	**28.00** ^**a**^ ** ± 1.00**	NA	NA	0.00 ± 0.00
*C. tropicalis*						
I_23_ Ct_1_^*^	0.00^c^ ± 0.00	13.33^b^ ± 0.88	**25.33** ^**a**^ ** ± 0.88**	NA	NA	9.33 ± 1.20

SE: Standard error, AMC 30: Amoxicillin-clavulanic acid, CIP 5: Ciprofloxacin, Flu 25: Fluconazole, NA: not applicable

Different letters indicate statistically significant differences (*p-value* < 0.05) between columns within the same test, highlighting the promising antimicrobial action of CMC-P NPs compared to both EEP and unloaded CMC NPs. A *p-value* < 0.05 was considered statistically significance for all analyses

##### MIC and MBC determination

Results presented in Table 3 demonstrated that Turkish EEP exhibited MIC values ranging from 0.105 mg/mL for *A. baumannii* and *Klebsiella spp.* to 7.5 mg/mL for *A. lwoffii* and *Serratia spp*., with MBCs varying from 0.325 to > 10 mg/mL. In addition, CMC NPs showed MIC and MBC values of 0.235–0.625 mg/mL for *Klebsiella* spp. and 0.938–1.25 mg/mL for *A. baumannii*, whereas *E. coli*,* H. alvei*,* P. aeruginosa*,* Serratia spp.*,* Staphylococcus* spp., and *Candida* spp. required > 10 mg/mL.

In comparison, CMC-P NPs displayed markedly enhanced antimicrobial activity, with MICs of 0.019–1.25 mg/mL and MBCs of 0.019–2.50 mg/mL against the tested MDR clinical microbial isolates. Notably, MDR *A. baumannii*,* K. pneumonia*, and *K. ozaenae* were the most sensitive (MIC; MBC = 0.019 mg/mL), followed by *Staphylococcus* spp. (MIC 0.039 mg/mL; MBC 0.078 mg/mL). *A. lwoffii*,* S. rubidaea*,* P. aeruginosa*, and *S. liquefaciens* were inhibited at 0.938–1.25 mg/mL and killed at 2.50 mg/mL, while *Candida* spp. showed MICs of 0.165–0.313 mg/mL and MBCs up to 0.625 mg/mL. Encapsulation of EEP within CMC NPs enhanced antimicrobial efficacy, reducing MIC values 1.2–8-fold compared to free EEP, with cleared bactericidal effects against *Klebsiella* spp., *A. baumannii*, and *Staphylococcus* spp. Collectively, CMC-P NPs demonstrated potent antimicrobial activity at substantially lower concentrations against MDR clinical microbial isolates.

**Table 3:** MIC and MBC determinations of EEP, CMC NPs, and CMC-P NPs on the most MDR clinical microbial isolates

Pathogen	Mean ± SE					
	CMC NPs (mg/mL)		EEP (mg/mL)		CMC-*P* NPs (mg/mL)	
	MIC	MBC	MIC	MBC	MIC	MBC
*A. lwoffii* I_9_ Al_1_^*^	> 10	> 10	7.50 ± 0.31	> 10	**1.25** ± 0.11	2.50 ± 0.15
*A. baumannii* I_87_ Ab_3_^*^	0.938 ± 0.18	1.25 ± 0.15	0.105 ± 0.01	0.325 ± 0.07	**0.019** ± 0.00	0.019 ± 0.001
*E. coli* I_93_ E_33_	> 10	> 10	0.75 ± 0.07	0.75 ± 0.11	**0.625** ± 0.11	1.25 ± 0.21
*H. alvei* I_65_ H_1_	> 10	> 10	0.50 ± 0.15	0.75 ± 0.07	**0.156** ± 0.008	0.156 ± 0.07
*K. pneumonia* I_33_ Kp_4_	0.235 ± 0.07	0. 625 ± 0.11	0.105 ± 0.008	0.325 ± 0.07	**0.019** ± 0.001	0.078 ± 0.00
*K. ozaenae* I_2_ Ko_1_^*^	0.235 ± 0.07	0. 625 ± 0.07	0.105 ± 0.01	0.325 ± 0.07	**0.019** ± 0.001	0.078 ± 0.00
*P. aeruginosa* I_37_ P_3_^*^	> 10	> 10	3.75 ± 0.22	7.5 ± 0.46	**0.938** ± 0.09	2.50 ± 0.22
*S. liquefaciens* I_38_ Sel_1_^*^	> 10	> 10	7.50 ± 0.46	> 10	**0.938** ± 0.11	2.50 ± 0.24
*S. rubidaea* I_48_ Ser_1_^*^	> 10	> 10	7.50 ± 0.31	> 10	**1.25** ± 0.19	2.50 ± 0.24
**Mean (Gram-negative bacteria)**	**> 10** ^**a**^	**> 10** ^**A**^	**3.091**^**b**^ **±1.64**	**> 10** ^**B**^	**0.579**^**c**^ **± 0.078**	**1.29** ^**C**^ **± 0.16**
*S. aureus* I_41_ St_2_^*^	> 10	> 10	0.185 ± 0.056	0.370 ± 0.006	**0.039** ± 0.00	0.078 ± 0.07
CON *Staphylococcus* I_84_ St_− 5_	> 10	> 10	0.185 ± 0.00	0.370 ± 0.04	**0.039** ± 0.008	0.078 ± 0.00
**Mean (Gram-positive bacteria)**	**> 10** ^**a**^	**> 10** ^**A**^	**0.185**^**b**^ **± 0.00**	**0.370**^**B**^ **± 0.00**	**0.039**^**c**^ **± 0.00**	**0.078**^**C**^ **± 0.00**
*C. albicans* I_72_ Ca_12_^*^	> 10	> 10	0.625 ± 0.11	0.750 ± 0.09	**0.313** ± 0.00	0.625 ± 0.09
*C. glabrata* I_66_ Cg_1_^*^	> 10	> 10	0.625 ± 0.09	1.25 ± 0.31	**0.313** ± 0.02	0.625 ± 0.11
*C. krusei* I_59_ Ck_1_	> 10	> 10	0.50 ± 0.07	0.750 ± 0.11	**0.165** ± 0.00	0.625 ± 0.07
*C. tropicalis* I_23_ Ct_1_^*^	> 10	> 10	0.625 ± 0.09	1.250 ± 0.22	**0.165** ± 0.008	0.625 ± 0.11
**Mean (Yeast)**	**> 10** ^**a**^	**> 10** ^**A**^	**0.594**^**b**^ **± 0.031**	**1.00**^**B**^ **± 0.12**	**0.239**^**c**^ **± 0.042**	**0.625**^**C**^ **± 0.00**

Different small letters indicate statistically significant differences (*p-value* < 0.05) between MIC mean values across different columns

Different capital letters indicate statistically significant differences (*p-value* < 0.05) between MBC mean values across different columns

Values > 10 were excluded from numerical ANOVA calculation but were assigned the highest significance letter category to reflect their minimal antimicrobial activity within the tested range

> 10: more than 10 mg/mL

#### Antimicrobial activity of honey encapsulated within CMC NPs

##### The agar-well diffusion method

Results illustrated in Table 4 and supplementary Plate 2 showed that Egyptian honey at a low concentration (10 mg/mL) exhibited no antimicrobial action against any microbial isolates under study. In contrast, CMC-H NPs demonstrated inhibitory effects against most MDR clinical isolates, with IZD values ranging 0.00–34.33 mm at the same concentration. The maximum bacterial inhibition was observed against *S. rubidaea* (IZD = 32.67 mm), followed by *Klebsiella* spp. (*K. ozaenae* and *K. pneumonia)*, CON *Staphylococcus*,* S. aureus*, S. *liquefaciens*, and *Acinetobacter* spp. with IZD values between 17.33 and 25.67 mm. However, *E. coli* and *H. alvei* were the least sensitive (IZD = 12.67–16.00 mm), while *P. aeruginosa* were not suppressed at this concentration. In addition, *Candida* spp. were inhibited by CMC-H NPs with IZD values ranging 17.33–34.33 mm, as *C. albicans* I_39_ Ca_7_ and *C. glabrata* I_66_ Cg_1_^*^ being the most sensitive yeast isolates.

Statistical analysis (p-values) demonstrated that CMC-H NPs possess significantly stronger antimicrobial activity than either honey or CMC NPs alone against all tested pathogens. This indicates that the encapsulation of honey within CMC NPs effectively enhances its antimicrobial efficacy, supporting the potential of CMC-H NPs as a therapeutic formulation.

**Table 4:** Antimicrobial activity of positive controls, honey, CMC NPs, CMC-H NPs at 10 mg/mL on MDR gram-negative, gram-positive bacteria, and yeast clinical isolates

Pathogen	Mean (mm) ± SE					
	CMC NPs	Honey	CMC-H NPs	AMC 30	CIP 5	Flu 25
*A. lwoffii*						
I_9_ Al_1_^*^	0.00^b^ ± 0.00	0.00^b^ ± 0.00	**17.33** ^**a**^ ** ± 0.67**	0.00 ± 0.00	0.00 ± 0.00	NA
*A. baumannii*						
I_73_ Ab_2_^*^	0.00^b^ ± 0.00	0.00^b^ ± 0.00	**17.33**^**a**^ **± 0.88**	0.00 ± 0.00	0.00 ± 0.00	NA
I_87_ Ab_3_^*^	13.67^b^ ± 0.33	0.00^c^ ± 0.00	**19.67**^**a**^ **± 1.20**	0.00 ± 0.00	9.33 ± 0.67	NA
I_88_ Ab_4_^*^	0.00^b^ ± 0.00	0.00^b^ ± 0.00	**19.00**^**a**^ **± 0.58**	0.00 ± 0.00	0.00 ± 0.00	NA
*E. coli*						
I_12_ E_6_	0.00^b^ ± 0.00	0.00^b^ ± 0.00	**12.67**^**a**^ **± 0.33**	0.00 ± 0.00	0.00 ± 0.00	NA
I_14_ E_8_	0.00^b^ ± 0.00	0.00^b^ ± 0.00	**15.33**^**a**^ **± 0.67**	0.00 ± 0.00	0.00 ± 0.00	NA
I_93_ E_34_	0.00^b^ ± 0.00	0.00^b^ ± 0.00	**16.00**^**a**^ **± 0.58**	0.00 ± 0.00	0.00 ± 0.00	NA
*H. alvei*						
I_65_ H_1_	0.00^b^ ± 0.00	0.00^b^ ± 0.00	**12.67**^**a**^ **± 0.33**	0.00 ± 0.00	0.00 ± 0.00	NA
*K. pneumoniae*						
I_33_ Kp_4_	19.33^b^ ± 0.33	0.00^c^ ± 0.00	**21.33**^**a**^ **± 0.67**	7.33 ± 0.33	7.00 ± 0.58	NA
I_56_ Kp_6_^*^	18.67^b^ ± 0.88	0.00^c^ ± 0.00	**20.33**^**a**^ **± 0.33**	0.00 ± 0.00	10.00 ± 0.00	NA
I_89_ Kp_8_^*^	18.00^b^ ± 0.58	0.00^c^ ± 0.00	**21.00**^**a**^ **± 0.58**	0.00 ± 0.00	0.00 ± 0.00	NA
*K. ozaenae*						
I_2_ Ko_1_^*^	18.00^b^ ± 1.15	0.00^c^ ± 0.00	**21.33**^**a**^ **± 0.67**	0.00 ± 0.00	0.00 ± 0.00	NA
I_80_ Ko_2_	17.33^b^ ± 0.88	0.00^c^ ± 0.00	**19.33**^**a**^ **± 0.88**	0.00 ± 0.00	8.33 ± 0.33	NA
I_83_ Ko_3_	18.00^b^ ± 0.58	0.00^c^ ± 0.00	**20.67**^**a**^ **± 0.33**	0.00 ± 0.00	7.67 ± 0.88	NA
I_85_ Ko_4_	17.67^b^ ± 0.33	0.00^c^ ± 0.00	**25.67**^**a**^ **± 0.88**	0.00 ± 0.00	0.00 ± 0.00	NA
*P. aeruginosa*						
I_37_ P_3_^*^	0.00^NA^ ± 0.00	0.00^NA^ ± 0.00	0.00^NA^ ± 0.00	0.00 ± 0.00	0.00 ± 0.00	NA
I_100_ P_8_	0.00^NA^ ± 0.00	0.00^NA^ ± 0.00	0.00^NA^ ± 0.00	0.00 ± 0.00	0.00 ± 0.00	NA
*S. liquefaciens*						
I_38_ Sel_1_^*^	0.00^b^ ± 0.00	0.00^b^ ± 0.00	**20.00**^**a**^ **± 0.58**	0.00 ± 0.00	0.00 ± 0.00	NA
*S. rubidaea*						
I_48_ Ser_1_^*^	0.00^b^ ± 0.00	0.00^b^ ± 0.00	**32.67**^**a**^ **± 1.45**	0.00 ± 0.00	0.00 ± 0.00	NA
CON *Staphylococcus* spp.						
I_84_ St_− 5_	0.00^b^ ± 0.00	0.00^b^ ± 0.00	**19.67**^**a**^ **± 0.33**	0.00 ± 0.00	0.00 ± 0.00	NA
I_92_ St_− 6_	0.00^b^ ± 0.00	0.00^b^ ± 0.00	**20.33**^**a**^ **± 0.33**	6.00 ± 0.58	8.33 ± 1.20	NA
*S. aureus*						
I_41_ St_2_^*^	0.00^b^ ± 0.00	0.00^b^ ± 0.00	**20.67**^**a**^ **± 0.33**	0.00 ± 0.00	9.67 ± 1.45	NA
I_44_ St_3_	0.00^b^ ± 0.00	0.00^b^ ± 0.00	**19.33**^**a**^ **± 0.67**	0.00 ± 0.00	0.00 ± 0.00	NA
*C. albicans*						
I_39_ Ca_7_	0.00^b^ ± 0.00	0.00^b^ ± 0.00	**34.33**^**a**^ **± 0.88**	NA	NA	9.33 ± 0.67
I_60_ Ca_8_^*^	0.00^b^ ± 0.00	0.00^b^ ± 0.00	**25.33**^**a**^ **± 1.33**	NA	NA	7.00 ± 0.58
I_72_ Ca_12_^*^	0.00^b^ ± 0.00	0.00^b^ ± 0.00	**19.67**^**a**^ **± 0.88**	NA	NA	0.00 ± 0.00
*C. glabrata*						
I_66_ Cg_1_^*^	0.00^b^ ± 0.00	0.00^b^ ± 0.00	**33.33**^**a**^ **± 0.88**	NA	NA	0.00 ± 0.00
*C. krusei*						
I_59_ Ck_1_	0.00^b^ ± 0.00	0.00^b^ ± 0.00	**19.67**^**a**^ **± 1.45**	NA	NA	0.00 ± 0.00
*C. tropicalis*						
I_23_ Ct_1_^*^	0.00^b^ ± 0.00	0.00^b^ ± 0.00	**17.33**^**a**^ **± 0.67**	NA	NA	9.33 ± 1.20

SE: Standard error, NA: not applicable, AMC 30: Amoxicillin-clavulanic acid, CIP 5: Ciprofloxacin, Flu 25: Fluconazole, NA: not applicable

Different letters indicate statistically significant differences (*p-value* < 0.05) between columns within the same test, highlighting the promising antimicrobial action of CMC-H NPs compared to both honey and unloaded CMC NPs. A *p-value* < 0.05 was considered statistically significance for all analyses

##### MIC and MBC determination

As shown in Table 5, the MIC values of Egyptian honey exceeded 10 mg/mL, ranging from 62.5 to 375 mg/mL against the fifteen MDR clinical microbial isolates. In contrast, the CMC-H NPs formulation demonstrated markedly enhanced antimicrobial efficacy, with MIC values ranging from 0.165 mg/mL to over 10 mg/mL. Obviously, *Klebsiella* spp. (*K. pneumonia* and *K. ozaenae*) were the most sensitive to CMC-H NPs, showing MIC and MBC values of 0.165 mg/mL, while *A. baumannii*, *S. rubidaea*, and *C. glabrata* exhibited MICs of 0.625 mg/mL and MBCs of 1.25–2.5 mg/mL. Gram-positive *Staphylococcus* spp. and most *Candida* spp. (except *C. glabrata*) were inhibited at MICs of 2.5 mg/mL and MBCs of 2.5–5 mg/mL. In comparison, *P. aeruginosa*, *E. coli*, and *H. alvei* required higher concentrations for inhibition, with MIC and MBC values of 7.5–>10 mg/mL. Overall, the close correspondence between MIC and MBC values for most pathogens indicates that CMC-H NPs exert not only strong growth-inhibitory effects but also potent bactericidal and fungicidal activity, particularly against *Klebsiella* spp., MDR Gram-positive bacteria, and yeast isolates.

**Table 5:** MIC and MBC determinations of Honey, CMC NPs, and CMC-H NPs on the most MDR clinical microbial isolates

Pathogen	Mean ± SE					
	CMC NPs (mg/mL)		Honey (mg/mL)		CMC-H NPs (mg/mL)	
	MIC	MBC	MIC	MBC	MIC	MBC
*A. lwoffii* I_9_ Al_1_^*^	> 10	> 10	> 10 (125)	> 10	**1.25** ± 0.23	1.25 ± 0.19
*A. baumannii* I_87_ Ab_3_^*^	0.938 ± 0.18	1.25 ± 0.15	> 10 (93.75)	> 10	**0.625** ± 0.09	1.25 ± 0.19
*E. coli* I_93_ E_33_	> 10	> 10	> 10 (125)	> 10	**7.50** ± 0.58	> 10
*H. alvei* I_65_ H_1_	> 10	> 10	> 10 (93.75)	> 10	**7.50** ± 0.58	> 10
*K. pneumonia* I_33_ Kp_4_	0.235 ± 0.07	0.625 ± 0.11	> 10 (375)	> 10	**0.165** ± 0.00	0.165 ± 0.03
*K. ozaenae* I_2_ Ko_1_^*^	0.235 ± 0.07	0.625 ± 0.11	> 10 (187.5)	> 10	**0.165** ± 0.00	0.165 ± 0.03
*P. aeruginosa* I_37_ P_3_^*^	> 10	> 10	> 10 (187.5)	> 10	**> 10**	> 10
*S. liquefaciens* I_38_ Sel_1_^*^	> 10	> 10	> 10 (125)	> 10	**1.25** ± 0.24	1.25 ± 0.16
*S. rubidaea* I_48_ Ser_1_^*^	> 10	> 10	> 10 (125)	> 10	**0.625** ± 0.11	1.25 ± 0.19
**Mean (Gram-negative bacteria)**	**> 10**	**> 10**	**> 10**	**> 10**	**> 10**	**> 10**
*S. aureus* I_41_ St_2_^*^	> 10	> 10	> 10 (62.5)	> 10	**2.50** ± 0.37	5.00 ± 0.00
CON *Staphylococcus* I_84_ St_− 5_	> 10	> 10	> 10 (125)	> 10	**2.50** ± 0.37	5.00 ± 0.27
**Mean (Gram-positive bacteria)**	**> 10** ^**a**^	**> 10** ^**A**^	**> 10** ^**a**^	**> 10** ^**A**^	**2.50**^**b**^ **± 0.00**	**5.00**^**B**^ **± 0.00**
*C. albicans* I_72_ Ca_12_^*^	> 10	> 10	--	--	**2.50** ± 0.31	5.00 ± 0.19
*C. glabrata* I_66_ Cg_1_^*^	> 10	> 10	--	--	**0.625** ± 0.11	2.5 ± 0.24
*C. krusei* I_59_ Ck_1_	> 10	> 10	--	--	**2.50** ± 0.19	5.00 ± 0.21
*C. tropicalis* I_23_ Ct_1_^*^	> 10	> 10	--	--	**2.50** ± 0.21	5.00 ± 0.00
**Mean (Yeast)**	**> 10** ^**a**^	**> 10** ^**A**^	**--**	**--**	**2.031**^**b**^ **± 0.46**	**3.75** ^**B**^ **± 0.72**

Different small letters indicate statistically significant differences (*p-value* < 0.05) between MIC mean values across different columns, while different capital letters indicate statistically significant differences (*p-value* < 0.05) between MBC pathogen mean values across different columns

Values > 10 were excluded from numerical ANOVA calculation but were assigned the highest significance letter category to reflect their minimal antimicrobial activity within the tested range

> 10: more than 10 mg/mL, --: not affected

#### Antimicrobial activity of royal jelly encapsulated within CMC NPs

##### The agar-well diffusion method

The data presented in Table 6 and supplementary Plate 3 indicated that Egyptian royal jelly, at a low concentration of 10 mg/mL, didn’t have any antimicrobial action against all 29 MDR clinical microbial isolates. In contrast, the encapsulated royal jelly within CMC NPs (CMC-RJ NPs) exhibited notable antimicrobial activity on most of the tested MDR isolates, with IZD values ranges between 0.00 and 30.33 mm at the same concentration. The CMC-RJ NPs formulation showed the highest antimicrobial activity against MDR strains of *E. coli*, *H. alvei*, *Klebsiella spp.* (*K. pneumoniae* and *K. ozaenae*), and *Serratia spp.* (*S. liquefaciens* and *S. rubidaea*), with IZD ranging 22.00–30.33 mm. Moreover, CMC-RJ NPs exhibited antimicrobial action on one isolate each of MDR *A. baumannii*, CON *Staphylococcus*, *S. aureus*, and *C. albicans*, as well as all *P. aeruginosa* isolates, with IZD values ranges between 14.67 and 22.00 mm. In comparison, *A. lwoffii* and non-albicans *Candida* isolates were not suppressed by CMC-RJ NPs at the tested concentration.

Statistical analysis (p-values) confirmed that CMC-RJ NPs exhibited significantly higher antimicrobial activity compared to royal jelly or CMC NPs alone across all tested microbial pathogens. These findings suggest that encapsulating royal jelly within CMC nanoparticles markedly improves its antimicrobial activity, reinforcing the potential of the CMC-RJ NPs formulation as a therapeutic agent.

**Table 6:** Antimicrobial activity of royal jelly, CMC NPs, and CMC-RJ NPs at 10 mg/mL on MDR gram-negative, gram-positive bacteria, and yeast clinical isolates

Pathogen	Mean (mm) ± SE					
	CMC NPs	Royal Jelly	CMC-RJ NPs	AMC 30	CIP 5	Flu 25
*A. lwoffii*						
I_9_ Al_1_^*^	0.00^NA^ ± 0.00	0.00^NA^ ± 0.00	0.00^NA^ ± 0.00	0.00 ± 0.00	0.00 ± 0.00	NA
*A. baumannii*						
I_73_ Ab_2_^*^	0.00^NA^ ± 0.00	0.00^NA^ ± 0.00	0.00^NA^ ± 0.00	0.00 ± 0.00	0.00 ± 0.00	NA
I_87_ Ab_3_^*^	13.67^b^ ± 0.33	0.00^c^ ± 0.00	**22.00**^**a**^ **± 1.00**	0.00 ± 0.00	9.33 ± 0.67	NA
I_88_ Ab_4_^*^	0.00^NA^ ± 0.00	0.00^NA^ ± 0.00	0.00^NA^ ± 0.00	0.00 ± 0.00	0.00 ± 0.00	NA
*E. coli*						
I_12_ E_6_	0.00^b^ ± 0.00	0.00^b^ ± 0.00	**26.67**^**a**^ **± 0.67**	0.00 ± 0.00	0.00 ± 0.00	NA
I_14_ E_8_	0.00^b^ ± 0.00	0.00^b^ ± 0.00	**24.00**^**a**^ **± 0.58**	0.00 ± 0.00	0.00 ± 0.00	NA
I_93_ E_34_	0.00^b^ ± 0.00	0.00^b^ ± 0.00	**25.00**^**a**^ **± 1.15**	0.00 ± 0.00	0.00 ± 0.00	NA
*H. alvei*						
I_65_ H_1_	0.00^b^ ± 0.00	0.00^b^ ± 0.00	**29.33**^**a**^ **± 0.88**	0.00 ± 0.00	0.00 ± 0.00	NA
*K. pneumoniae*						
I_33_ Kp_4_	20.33^b^ ± 0.33	0.00^c^ ± 0.00	**30.33**^**a**^ **± 1.20**	7.33 ± 0.33	7.00 ± 0.58	NA
I_56_ Kp_6_^*^	18.67^b^ ± 0.88	0.00^c^ ± 0.00	**27.33**^**a**^ **± 0.33**	0.00 ± 0.00	10.00 ± 0.00	NA
I_89_ Kp_8_^*^	18.00^b^ ± 0.58	0.00^c^ ± 0.00	**25.33**^**a**^ **± 0.33**	0.00 ± 0.00	0.00 ± 0.00	NA
*K. ozaenae*						
I_2_ Ko_1_^*^	18.00^b^ ± 1.15	0.00^c^ ± 0.00	**28.00**^**a**^ **± 0.58**	0.00 ± 0.00	0.00 ± 0.00	NA
I_80_ Ko_2_	17.33^b^ ± 0.88	0.00^c^ ± 0.00	**27.00**^**a**^ **± 0.58**	0.00 ± 0.00	8.33 ± 0.33	NA
I_83_ Ko_3_	18.00^b^ ± 0.58	0.00^c^ ± 0.00	**25.67**^**a**^ **± 0.33**	0.00 ± 0.00	7.67 ± 0.88	NA
I_85_ Ko_4_	17.67^b^ ± 0.33	0.00^c^ ± 0.00	**24.67**^**a**^ **± 0.88**	0.00 ± 0.00	0.00 ± 0.00	NA
*P. aeruginosa*						
I_37_ P_3_^*^	0.00^b^ ± 0.00	0.00^b^ ± 0.00	**14.67**^**a**^ **± 0.88**	0.00 ± 0.00	0.00 ± 0.00	NA
I_100_ P_8_	0.00^b^ ± 0.00	0.00^b^ ± 0.00	**15.33**^**a**^ **± 0.88**	0.00 ± 0.00	0.00 ± 0.00	NA
*S. liquefaciens*						
I_38_ Sel_1_^*^	0.00^b^ ± 0.00	0.00^b^ ± 0.00	**24.00**^**a**^ **± 0.58**	0.00 ± 0.00	0.00 ± 0.00	NA
*S. rubidaea*						
I_48_ Ser_1_^*^	0.00^b^ ± 0.00	0.00^b^ ± 0.00	**22.00**^**a**^ **± 0.58**	0.00 ± 0.00	0.00 ± 0.00	NA
CON *Staphylococcus* spp.						
I_84_ St_− 5_	0.00^b^ ± 0.00	0.00^b^ ± 0.00	**18.33**^**a**^ **± 0.67**	0.00 ± 0.00	0.00 ± 0.00	NA
I_92_ St_− 6_	0.00^NA^ ± 0.00	0.00^NA^ ± 0.00	0.00^NA^ ± 0.00	6.00 ± 0.58	8.33 ± 1.20	NA
*S. aureus*						
I_41_ St_2_^*^	0.00^b^ ± 0.00	0.00^b^ ± 0.00	**19.00**^**a**^ **± 0.58**	0.00 ± 0.00	9.67 ± 1.45	NA
I_44_ St_3_	0.00^NA^ ± 0.00	0.00^NA^ ± 0.00	0.00^NA^ ± 0.00	0.00 ± 0.00	0.00 ± 0.00	NA
*C. albicans*						
I_39_ Ca_7_	0.00^b^ ± 0.00	0.00^b^ ± 0.00	**17.67**^**a**^ **± 0.33**	NA	NA	9.33 ± 0.67
I_60_ Ca_8_^*^	0.00^NA^ ± 0.00	0.00^NA^ ± 0.00	0.00^NA^ ± 0.00	NA	NA	7.00 ± 0.58
I_72_ Ca_12_^*^	0.00^NA^ ± 0.00	0.00^NA^ ± 0.00	0.00^NA^ ± 0.00	NA	NA	0.00 ± 0.00
*C. glabrata*						
I_66_ Cg_1_^*^	0.00^NA^ ± 0.00	0.00^NA^ ± 0.00	0.00^NA^ ± 0.00	NA	NA	0.00 ± 0.00
*C. krusei*						
I_59_ Ck_1_	0.00^NA^ ± 0.00	0.00^NA^ ± 0.00	0.00^NA^ ± 0.00	NA	NA	0.00 ± 0.00
*C. tropicalis*						
I_23_ Ct_1_^*^	0.00^NA^ ± 0.00	0.00^NA^ ± 0.00	0.00^NA^ ± 0.00	NA	NA	9.33 ± 1.20

Different letters indicate statistically significant differences (*p-value* < 0.05) between columns within the same test, highlighting the promising antimicrobial action of CMC-RJ NPs compared to both royal jelly and unloaded CMC NPs. A *p-value* < 0.05 was considered statistically significance for all analyses. NA: not applicable

##### MIC and MBC determination

Based on Table 7, Egyptian royal jelly exhibited MICs and MBCs > 10 mg/mL against fifteen MDR clinical isolates. In contrast, CMC-RJ NPs formulation showed markedly improved antimicrobial activity, with MIC values ranging from 0.078 to > 10 mg/mL. Notably, *Klebsiella* spp. (*K. pneumonia* and *K. ozaenae*) were the most susceptible, with MIC and MBC of 0.078 mg/mL, followed by *E. coli* and *H. alvei*, with MIC and MBC value of 0.313 mg/mL. *A. baumannii*,* S. liquefaciens*, and *S. rubidaea* showed MICs of 0.625–1.25 mg/mL and MBCs of 1.25 mg/mL. Gram-positive *Staphylococcus* spp. were inhibited at 2.5 mg/mL and killed at 5 mg/mL. *P. aeruginosa*, *A. lwoffii*, and *Candida* spp. required higher concentrations (MICs 7.5–>10 mg/mL; MBCs > 10 mg/mL). Overall, lower concentrations of CMC-RJ NPs were effective on MDR Gram-negative bacteria (except *P. aeruginosa*), while higher concentrations were required to kill MDR *P. aeruginosa*, Gram-positive bacteria, and *Candida* spp.

**Table 7:** MIC and MBC determinations of royal jelly, CMC NPs, and CMC-RJ NPs on the most MDR clinical microbial isolates

Pathogen	Mean ± SE					
	CMC NPs (mg/mL)		Royal Jelly (mg/mL)		CMC-RJ NPs (mg/mL)	
	MIC	MBC	MIC	MBC	MIC	MBC
*A. lwoffii* I_9_ Al_1_^*^	> 10	> 10	> 10 (46.88)	> 10 (93.76)	> 10	> 10
*A. baumannii* I_87_ Ab_3_^*^	0.938 ± 0.18	1.25 ± 0.15	> 10 (23.43)	> 10 (23.43)	**0.625** ± 0.09	1.25 ± 0.19
*E. coli* I_93_ E_33_	> 10	> 10	> 10 (23.43)	> 10 (46.88)	**0.313** ± 0.05	0.313 ± 0.009
*H. alvei* I_65_ H_1_	> 10	> 10	> 10 (17.1)	> 10 (46.88)	**0.313** ± 0.11	0.313 ± 0.07
*K. pneumonia* I_33_ Kp_4_	0.235 ± 0.07	0.625 ± 0.11	> 10 (46.88)	> 10 (46.88)	**0.078** ± 0.006	0.078 ± 0.00
*K. ozaenae* I_2_ Ko_1_^*^	0.235 ± 0.07	0.625 ± 0.11	> 10 (46.88)	> 10 (46.88)	**0.078** ± 0.00	0.078 ± 0.00
*P. aeruginosa* I_37_ P_3_^*^	> 10	> 10	> 10 (23.43)	> 10 (46.88)	**7.50** ± **0.58**	> 10
*S. liquefaciens* I_38_ Sel_1_^*^	> 10	> 10	> 10 (46.88)	> 10 (93.76)	**0.938** ± **0.17**	1.25 ± 0.14
*S. rubidaea* I_48_ Ser_1_^*^	> 10	> 10	> 10 (46.88)	> 10 (93.76)	**1.25** ± **0.19**	1.25 ± 0.19
**Mean (Gram-positive bacteria)**	**> 10**	**> 10**	**> 10**	**> 10**	**> 10**	**> 10**
*S. aureus* I_41_ St_2_^*^	> 10	> 10	> 10 (11.50)	> 10 (23.43)	**2.5** ± **0.31**	5.00 ± 0.35
CON *Staphylococcus* I_84_ St_− 5_	> 10	> 10	> 10 (17.1)	> 10 (23.43)	**2.5** ± **0.31**	5.00 ± 0.35
**Mean (Gram-negative bacteria)**	**> 10** ^**a**^	**> 10** ^**A**^	**> 10** ^**a**^	**> 10** ^**A**^	**2.50**^**b**^ **± 0.00**	**5.00**^**B**^ **± 0.00**
*C. albicans* I_72_ Ca_12_^*^	> 10	> 10	--	--	> 10	> 10
*C. glabrata* I_66_ Cg_1_^*^	> 10	> 10	--	--	> 10	> 10
*C. krusei* I_59_ Ck_1_	> 10	> 10	--	--	> 10	> 10
*C. tropicalis* I_23_ Ct_1_^*^	> 10	> 10	--	--	> 10	> 10
**Mean (Yeast)**	**> 10**	**> 10**	**--**	**--**	**> 10**	**> 10**

Different small letters indicate statistically significant differences (*p-value* < 0.05) between MIC mean values across different columns, while different capital letters indicate statistically significant differences (*p-value* < 0.05) between MBC mean values across different columns

Values > 10 were excluded from numerical ANOVA calculation but were assigned the highest significance letter category to reflect their minimal antimicrobial activity within the tested range

> 10: more than 10 mg/mL, --: not affected

### Cell viability analysis of bee product-loaded CMC NPs via MTT assay

**Fig. 5:**
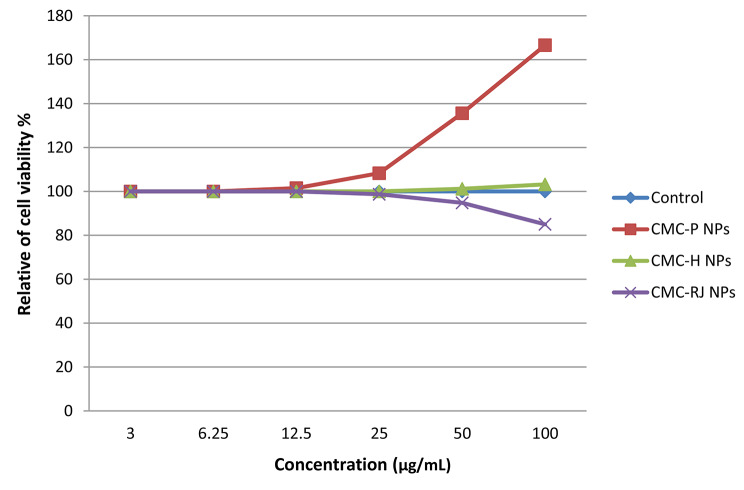
Cell viability of RPEl cells treated with different concentrations of CMC-P NPs, CMC-H NPs, and CMC-RJ NPs as assessed by MTT assay

The effect of CMC-P NPs, CMC-H NPs, and CMC-RJ NPs on the cell viability of normal RPE1 cells was evaluated using the MTT assay, as presented in Table 8; Fig. 5. Treatment with CMC-P NPs at 50 and 100 µg/mL resulted in a significant increase in cell viability, reaching 135.6% and 166.6% relative to the untreated control (set at 100%), respectively. At concentrations ≤ 25 µg/mL, a non-obvious no significant change in cell viability was observed. These findings indicate that CMC-P NPs exhibit no cytotoxic effects and may enhance cell viability under the tested conditions.

Further, according to Table 7; Fig. 5, treatment with CMC-H NPs at concentrations of 50 and 100 µg/mL resulted in a slight increase in cell proliferation, while concentrations below 50 µg/mL did not affect cell growth. On the other hand, CMC-RJ NPs exhibited no effect on cell viability at low concentrations (3, 6.25, and 12.5 µg/mL), with a recorded cell viability of 100%. However, higher concentrations (25, 50, and 100 µg/mL) slightly decreased cell viability to 98.7%, 94.8%, and 85%, respectively, confirming the preliminary biosafety and biocompatibility of the prepared nano-formulation.

**Table 8:** Effect of CMC-based nano-formulations on RPE1 cell viability (%)

Concentration (µg/mL)	Mean cell viability (%) ± SE			
	Control	CMC-*P* NPs	CMC-H NPs	CMC-RJ NPs
3	100^a^ ± 0.00	100^a^ ± 0.00	100^a^ ± 0.00	100^c^ ± 0.00
6.25	100^a^ ± 0.00	100^a^ ± 0.00	100^a^ ± 0.00	100^c^ ± 0.00
12.5	100^a^ ± 0.00	101.5^a^ ± 2.1	100^a^ ± 0.00	100^c^ ± 0.00
25	100^a^ ± 0.00	108.3^a^ ± 1.4	100^a^ ± 0.00	98.7^bc^ ± 1.7
50	100^a^ ± 0.00	135.6^b^ ± 3.2	101.2^a^ ± 0.8	94.8^b^ ± 1.4
100	100^a^ ± 0.00	166.6^c^ ± 5.8	103.2^a^ ± 3.1	85.00^a^ ± 2.3

Values are expressed as mean ± SE (*n* = 3)

Cell viability is presented relative to untreated control (100%)

Different small letters within the same column indicate statistically significant differences (*p-value* < 0.05) between concentrations (across rows) for each formulation

## Discussion

In the current study, the qualitative determination of polyphenols was undertaken using HPLC analysis to identify the bioactive components of bee products (Turkish EEP, Egyptian honey, and Egyptian royal jelly). The HPLC chromatographic analysis of Turkish EEP revealed a diverse polyphenol profile comprising 18 compounds, with daidzein and naringenin being the most abundant. Other detected compounds included hesperetin, caffeic acid, apigenin, coumaric acid, cinnamic acid, kaempferol, methyl gallate, querctin, and ellagic acid, while Pyro catechol and catechin were present in minor amounts. Previous studies have shown the phenolic content of propolis varies significantly depending on geographic region, plant origin, and collection season [40–42]. Altuntaş et al. [43] reported cinnamic acid levels (0.88–7.38 mg/g) in Turkish propolis extract, similar to our findings, while their caffeic acid levels (0.24–7.84 mg/g) were lower than our results. Özkök et al. [40] identified lower amounts of caffeic acid (4.03 mg/g), p-coumaric acid (2.33 mg/g), cinnamic acid (2.26 mg/g), apigenin (1.96 mg/g), and galangin (1.07 mg/g) in Turkish propolis extract from Adana, while quercetin and ferulic acid were not detected.

Similarly, the HPLC chromatogram of Egyptian honey revealed the presence of 7 polyphenols. Indeed, Egyptian honey showed a high content of chlorogenic acid and lower amounts of gallic acid, caffeic acid, naringenin, ferulic acid, rutin, and daidzein. These findings are consistent with those of Smetanska et al. [44], who reported chlorogenic acid as the predominant phenolic acid in various honey types from different countries. Likewise, Halawani [45] found the highest polyphenol contents in Saudi honey were chlorogenic acid and gallic acid. On the other hand, pinocembrin and chrysin were the major components in Northern Cyprus honey, according to Uçar et al. [46]. These variations are likely attributed to different geographic origins and floral sources.

Furthermore, the HPLC chromatogram of Egyptian royal jelly revealed the presence of 13 polyphenols. Daidzein and querctin were the most predominant constituents, followed by hesperetin, caffeic acid, and gallic acid. Other compounds were in minor amounts and included chlorogenic acid, vanillin, apigenin, cinnamic acid, coumaric acid, and methyl gallate. Previous studies by López-Gutiérrez et al. [47] and Oršolić and Jazvinšćak Jembrek [48] reported hesperetin, apigenin, naringenin, and ferulic acid in Spain’s royal jelly in different concentrations depending on the botanical origins. Further, gallic acid, apigenin, quercetin, caffeic acid, ellagic acid, vanillin, rutin, chlorogenic acid, and kaempferol were the primary polyphenols found in Bingol royal jelly [49].

Notably, several identified polyphenols are known for their potent biological activities. Naringenin and its derivatives exhibit antimicrobial activity, particularly against MRSA infections, by affecting membrane fluidity, binding DNA, and reducing toxin production and biofilm formation in MDR bacteria [50]. Both naringenin and daidzein also have potent anti-inflammatory and antioxidant properties and they are used in tumor treatment [51]. High levels of kaempferol and p-coumaric acid contribute to antibacterial activity against *S. aureus*, *S. saprophyticus*, and *Enterobacter faecalis*, along with anti-inflammatory and antioxidant activity [52]. Cinnamic acid and its derivatives inhibit bacteria by rupturing cell membranes and preventing cell division, ATPase enzymes, and biofilm formation [53]. Further, chlorogenic acid has significant roles as an antibacterial against Gram-positive and Gram-negative bacteria, and as an antiviral, antioxidant, hepatoprotective, and anti-inflammatory agent [54, 55]. Gallic acid, likewise, demonstrates strong antimicrobial activity, particularly against Gram-negative microbes [56].

The particle size and distribution of the prepared formulations were assessed using DLS measurements. This technique is widely used to study the stability of the nanostructures and detect any signs of aggregation or agglomeration [57]. Particle size a critical characteristic for applications of nanoparticles especially in biomedical fields [58]. Cellular uptake of nanoparticles is strongly affected by their physicochemical properties, such as size and surface charge. Smaller particles possess a larger surface area, promoting their ability to adhere to cell surfaces and facilitate drug release. This strong adhesion indicates a greater chance for nanoparticles to be internalized by cells [59]. According to our findings, the average particle sizes of CMC NPs, CMC-P NPs, CMC-RJ NPs, and CMC-H NPs were 12.16, 100.7, 134.5, and 350.8 nm, respectively. These nano-formulations exhibited particle sizes within the optimal range of 10–1000 nm, which is suitable for various biomedical applications. Submicron-sized particles can be used as drug carriers with controlled-release targets, such that the drug acts directly at the destination [60]. Moreover, nanoparticles with smaller diameters are known to improve the potency, solubility, and bioavailability of active components in formulations [61]. In line with our results, previous studies by **Nguyen et al.** [11] and **Zhang et al.** [62] prepared CMC NPs and propolis-loaded zein/CMC NPs with particle sizes of 32.68 nm and 156 nm, respectively. The obtained CMC-P NPs, CMC-H NPs, and CMC-RJ NPs in smaller nano-sizes make them good candidates for antimicrobial applications, as their larger surface area enhances interaction with microbial cells, leading to more effective bactericidal effects than larger particles. Also, the small PDI values in this study indicate a homogenous size distribution of nanoparticles and a tendency for aggregation [63]. Similarly, **Rizvi et al.** [39] prepared CMC-nanosponges with a particle size of 195 nm and a PDI of 0.279, comparable to our results.

Zeta potential is an important characterization technique used to assess the surface charge of nanoparticles and predict their colloidal stability [64]. Generally, nanoparticles with zeta potential values above ± 30 mV are considered physically stable, as electrostatic repulsion between similarly charged nanoparticles prevents aggregation in suspension [65]. In the present study, the CMC NPs exhibited a significant a negative zeta potential (-35.8 mV), primarily due to the ionization of negatively charged carboxyl groups (COO^− e^ on their surface [13]. Similarly, the observed negative zeta potential value of EEP (-28.2 mV) is attributed to the presence of ionized polyphenol functional groups, a result consistent with the findings of Hegazi et al. [66]. The negative zeta potential of Egyptian honey (-11.9 mV) can be explained by its high sugar contents and organic acids, which were partially ionized in in aqueous solution to yield carboxylate anions [67]. This observation was supported by Clebis et al. [68], who reported zeta potential values for Brazilian honey ranging between–3.8 and − 0.436 mV. On the other hand, Egyptian royal jelly exhibited a slightly positive zeta potential, likely due to the presence of protonated amino groups of its major proteins [69]. Following encapsulation, a remarkable shift in zeta potential was observed. The values for CMC-P NPs, CMC-H NPs, and CMC-RJ NPs dropped significantly to -67, -65.5, and − 45.0 mV, respectively. This shift can be attributed to the dominant influence of the anionic CMC matrix at the nanoparticle surface after encapsulation, together with the incorporation of negatively charged bioactive constituents such as phenolic compounds from EEP and honey, which may enhance the overall surface charge density [70]. Such increased negative charge contributes to stronger electrostatic repulsion between nanoparticles, thereby improving colloidal stability [66]. Hence, these changes indicate the successful incorporation of EEP, honey, and royal jelly within CMC NPs and the enhanced stability of the prepared nano-formulations, which make them plausible candidates for biomedical applications [62, 66].

FTIR spectroscopy is a good characterization technique used to study the potential interactions between nano-polymer (CMC NPs) and bee products during the encapsulation process. Such interactions are indicated by shifts in the characteristic peaks of the nanoparticles after encapsulation [71]. In our study, the FTIR spectra of EEP, honey, royal jelly, and CMC NPs showed their characteristic peaks. Also, the spectra of loaded nanoparticles (CMC-P NPs, CMC-H NPs, and CMC-RJ NPs) retained the characteristic bands of the CMC NPs, indicating that the encapsulation of bee products did not alter the polymer structure. These confirmed that the bee products were physically entrapped within the CMC NPs without any significant chemical interactions, confirming their stability and compatibility with the polymer during the encapsulation process [39].

The antimicrobial activity bee products, CMC NPs, and their respective loaded nanoparticles (CMC-P NPs, CMC-H NPs, and CMC-RJ NPs) were determined using the agar-well diffusion method and MIC determination. At a concentration of 10 mg/mL, Turkish EEP showed bactericidal activity against all 29 MDR isolates (IZD: 9.33–20.33 mm; MIC: 0.105–7.5 mg/mL; MBC: 0.325–>10 mg/mL), while CMC NPs were only effective against *Klebsiella* spp. and one *A. baumannii* isolate (IZD: 13.67–20.33 mm; MIC: 0.235–>10 mg/mL; MBC: 0.625–>10 mg/mL). However, when EEP was encapsulated into CMC NPs, the resulting CMC-P NPs exhibited significantly enhanced antimicrobial activity and remarkely low MIC values (IZD: 17.67–31.33 mm; MIC: 0.019–1.25 mg/mL; MBC: 0.019–2.5 mg/mL) against all tested isolates. These findings are in line with previous studies by Gaber et al. [72] and Segueni et al. [73], who reported the effectiveness of Turkish EEP against *S. aureus*, *P. aeruginosa*, *A. baumannii*, *C. albicans*, and *C. glabrata* at low concentrations. The antimicrobial action of propolis is mainly attributed to its high content of phenolic acids and flavonoids [42]. Similarly, Ong et al. [61] reported the antimicrobial efficacy of chitosan-propolis nano-formulation against MDR *Enterococcus faecalis*. Further, Soliman et al. [74] reported that lectin-conjugated chitosan nanoparticles possess a good antibacterial activity against *Enterococcus faecalis*, *Salmonella typhimurium*, *Shigella sonnei*, and *S. aureus*. Interestingly, our CMC-P NPs achieved a MIC of 0.019 mg/mL, which is notably lower (more potent) than the 0.062 mg/mL reported by Elnaggar et al. [75] for propolis-colistin-integrated chitosan nanoparticles on colistin-resistant *K. Pneumonia*, highlighting the good potential of the prepared formulations. Furthermore, a recent study by Dumitru et al. [17] supports the synergistic potential of combining bee products within biopolymer matrices. While they reported inhibition zones of up to 10 mm using honey, propolis, and royal jelly in alginate–chitosan films, our CMC-based nanoparticles achieved significantly broader zones (up to 34.33 mm).

On the other hand, Egyptian honey at 10 mg/mL concentration showed no antimicrobial activity (IZD = 0.00 mm; MIC > 10 mg/mL), as previously described by Helmy et al. [26]. However, its respective nano-formulations (CMC-H NPs) demonstrated notable antimicrobial activity but the inhibition varied according to the pathogen itself, with IZD values ranging from 0.00 to 34.33 mm as well as MIC and MBC values between 0.165 and > 10 mg/mL. These findings highlight the improved antimicrobial activity of CMC-H NPs compared to pure honey and CMC NPs. Similarly, Noori et al. [76] reported the antibacterial action of honey-loaded PVA/chitosan/montmorillonite nanocomposite against *S. aureus* from wound infection, where the presence of honey in the nanocomposite improved its antibacterial activity.

Moreover, Egyptian royal jelly at a concentration of 10 mg/mL showed no antimicrobial activity and reported MIC values over 10 mg/mL, as previously described by Helmy et al. [26]. However, its respective nano-formulations (CMC-RJ NPs) formulation demonstrated significantly enhanced antimicrobial efficacy, with MICs and MBCs ranging from 0.078 to > 10 mg/mL depending on the pathogen. Moreover, MIC and MBC findings revealed that lower concentrations of CMC-RJ NPs were effective on MDR Gram-negative bacteria, while higher concentrations were required to kill MDR Gram-positive bacteria and *Candida* spp. This difference in susceptibility may be attributed to variations in cell wall structure among Gram-negative, Gram-positive bacteria, and *Candida* spp. Gram-positive bacteria possess a thick, multilayered peptidoglycan wall, while Gram-negative bacteria have a thin peptidoglycan layer and an outer membrane rich in lipopolysaccharides [77, 78], which may affect permeability and interaction with the nanoparticles.

Recently, the emergence and rapid spread of new resistant clinical pathogens has dramatically increased the global threat generated by microbial infections [3]. Encapsulation of antimicrobial agents within nano-polymers has great potential in enhancing their antimicrobial potency and overcoming the growing challenge of antibiotic resistance. These nano-formulations offer several advantages, including controlled release, targeted delivery, protecting active components from decomposition or inactivation, improved bioavailability, and reduced toxicity to non-target tissues [77].

Consequently, the enhanced antimicrobial efficacy observed following the encapsulation of bee products within CMC nanoparticles may be attributed to the synergistic interaction between the bioactive constituents of these natural products and the physicochemical properties of the CMC NPs. The improved inhibition of microbial growth by CMC-P NPs, CMC-H NPs, and CMC-RJ NPs, compared to their pure compounds, may be related to the presence of phenolics and flavonoids in propolis, hydrogen peroxide, and polyphenols in honey, and bioactive peptides and phenolic compounds in royal jelly. These bioactive components have previously been reported to exhibit enhanced antimicrobial activity when incorporated into chitosan-based nanocarriers, suggesting a possible synergistic effect between natural antimicrobial compounds and nanoparticle systems [79]. In addition, the antimicrobial performance of nanoparticles is influenced by key physicochemical parameters such as particle size and surface charge [77, 80]. Surface charge may facilitate nanoparticle–microbial membrane interactions, potentially increasing membrane permeability and promoting oxidative stress mechanisms including reactive oxygen species (ROS) generation [81, 82]. Supporting these mechanisms, **El-Houssiny et al.** [83] reported increased membrane permeability of *S. aureus* upon treatment with negatively charged Alginate-Moringa nanoparticles. In addition to the previously discussed pathways, nanoparticles have been reported to exert antimicrobial effects through multiple mechanisms, including direct interaction with microbial cells, inhibition of biofilm formation, and intracellular interference with cellular components such as DNA or proteins [80, 84]. Taken together, these findings suggest that similar physicochemical interaction pathways may contribute to the enhanced activity observed in the present formulations. However, direct mechanistic validation was beyond the scope of this study.

In this study, we utilized the advantages of nano-formulations to enhance the efficiency of natural antimicrobial agents, overcome drug-resistant pathogens, and find alternatives to conventional antibiotics. Obviously, the three prepared nano-formulation systems demonstrated significant antimicrobial action on MDR pathogens, with particularly strong effects against *K. pneumonia* and *K. ozaenae*, which have resisted most antibiotic classes in this study. Among the three systems, CMC-P NPs emerged as the most potent formulation, exhibiting bactericidal action against all tested bacterial and yeast strains, demonstrating superior effectiveness compared to CMC-H NPs and CMC-RJ NPs.

Assessment of cell viability was carried out using the MTT assay to ensure the safety and biocompatibility of the developed nano-formulations in biomedical applications. MTT is a colorimetric assay to measure the activity of cellular enzymes that reduce the yellow tetrazolium dye (MTT) to insoluble purple formazan crystals in living cells [85]. The MTT assay results demonstrated that all three nano-formulations (CMC-P NPs, CMC-H NPs, and CMC-RJ NPs) were non-toxic to normal RPE1 cells.

Indeed, CMC-P NPs significantly enhanced cell proliferation at concentration ≥ 50 µg/mL, with an increase reaching up to 66% over the control. This proliferative effect may be attributed to bioactive polyphenols in propolis extract, which are known for their potent antioxidant activity and regenerative potential [42, 86]. These compounds have a critical role in protecting against oxidative stress and have been associated with tissue regeneration and healing in dermatological applications, such as eczema, ulcers, burn wounds, and membrane infections [87, 88]. The present findings are consistent with Ozdal et al. [89], who reported a similar effect using Turkish propolis on normal fibroblasts and mesenchymal stem cells. Additionally, studies by Fung et al. [90] and Elkhenany et al. [91] confirmed the proliferative effect of propolis on stem cells from human-exfoliated deciduous teeth and mouse bone marrow, respectively. Accordingly, our results demonstrated that the EEP of Turkish propolis encapsulated within CMC nanoparticles significantly supports the proliferation of normal RPE1 cells, suggesting its potential as a safe and effective formulation for biomedical applications.

For CMC-H NPs, a slight increase in RPE1 cell proliferation was observed at concentrations of 50–100 µg/mL, which may be attributed to the metabolic stimulation provided by honey sugars, acting as an energy source [92, 93]. These observations are supported by Al-Jadi et al. [92], who reported a similar stimulatory effect of Malaysian honey on fibroblast proliferation.

In the case of CMC-RJ NPs, no adverse effects on cell viability were detected at low concentrations (3–12.5 µg/mL). A minimal reduction in cell viability was noted at higher concentrations (25–100 µg/mL), with cell viability remaining within a safe range (≥ 85%). According to the American National Cancer Institute guidelines, a compound is cytotoxic if its IC_50_ is less than 30 µg/mL [94, 95]. Since the IC_50_ for CMC-RJ NPs in the current study was greater than 100 µg/mL, the CMC-RJ NPs formulation can be regarded a preliminary non-toxic and biocompatible within the tested concentration range.

The findings provide a comparative view of how different bee-derived bioactive matrices behave within the same CMC-based nano-delivery system. The observed differences in antimicrobial performance, particularly with CMC-P NPs, highlight the value of evaluating multiple natural products within a unified platform. As a preliminary investigation, these results warrant further optimization and validation before potential therapeutic application.

## Conclusion and study limitations

This study demonstrates that nano-encapsulation of bee products within CMC NPs significantly enhances their antimicrobial activity against MDR clinical pathogens. Among the developed formulations, EEP-loaded CMC nanoparticles (CMC-P NPs) exhibited superior antimicrobial efficacy, showing broad-spectrum bactericidal activity against all tested MDR isolates and the lowest MIC and MBC values. The higher polyphenolic content of EEP, as confirmed by HPLC analysis, may contribute to this enhanced antimicrobial performance.

The prepared nano-formulations exhibited nanoscale particle sizes, favorable physicochemical properties, and promising preliminary cytocompatibility toward normal RPE1 cells. These findings highlight the potential of CMC-based bee product nano-formulations as antimicrobial delivery systems for future biomedical applications. Additionally, they emphasize the value of evaluating multiple natural products within a unified platform. As an initial investigation, these results warrant further optimization and validation prior to any potential therapeutic application.

Despite these promising in vitro findings, the present study remains limited by its experimental scope. All investigations were conducted under in vitro conditions, which may not fully reflect the behavior of the nano-formulations in complex physiological environments. In addition, molecular mechanisms underlying the enhanced antimicrobial activity were not explored at this stage. Furthermore, certain physicochemical parameters—including encapsulation efficiency (EE), loading capacity (LC), and release kinetics—as well as long-term stability and cytotoxicity evaluation on cancer cell lines, were beyond the scope of this work. Further studies, including in vivo validation and mechanistic investigations, are warranted to strengthen the translational potential of the developed nano-formulations.

## Electronic Supplementary Material

Below is the link to the electronic supplementary material.

Supplementary Material 1

## Data Availability

All information created or analyzed during the present study are included in the manuscript.

## Abbreviations

CMC NPs: Carboxymethyl chitosan nanoparticles
CMC-H NPs: Carboxymethyl chitosan – Honey nanoparticles
CMC-P NPs: Carboxymethyl chitosan–propolis extract nanoparticles
CMC-RJNPs: Carboxymethyl chitosan– Royal Jelly nanoparticles
IZD: Inhibition zone diameter
MDR: Multidrug-resistant
MBC: Minimum bactericidal concentration
MIC: Minimum inhibitory concentration
PDI: Polydispersity index
ROS: Reactive oxygen species
RPE1: Retinal pigment epithelial
SE: Standard error
