# Supplementary material for: Antimicrobial potential of different bee product-loaded carboxymethyl chitosan nanoparticles against multidrug-resistant clinical pathogens: a comparative in vitro study
Source: BMC Biotechnol. 2026 May 29;26:71. doi: 10.1186/s12896-026-01159-5 (PMC13220480; doi:10.1186/s12896-026-01159-5)
Supplement: Supplementary file 1 — Supplementary Material 1 [file 12896_2026_1159_MOESM1_ESM.docx]

**Supplementary Table (S1):** List of MDR clinical microbial isolates from different infections and their resistance to different antimicrobials

| **Clinical isolate** | **Infection source** | **Resistance ratio** | **Resistant antibiotics** | **Sensitive antibiotics** |
| --- | --- | --- | --- | --- |
| *A. baumannii* I_73_ Ab_2_^*^ | Chest | 23/25 | P 10, AMP 10, AMC 30, SAM 20, CAZ 30, CFM 5, CFP 75, CRO 30, CTX 30, FEP 30, CIP 5, NOR 10, OFX 5, AK 30,CN 10, CL 30, ETP 10, MEM 10, CZA 50, C/T, SCF 105, SXT 25, and TZP 110 | TGC 15 and CT 10 |
| *A. baumannii* I_87_ Ab_3_^*^ | Chest | 23/25 | P 10, AMP 10, AMC 30, SAM 20, CAZ 30, CFM 5, CFP 75, CRO 30, CTX 30, FEP 30, CIP 5, NOR 10, OFX 5, AK 30, CN 10, CL 30, ETP 10, MEM 10, CT 10, C/T, SCF 105, SXT 25, and TZP 110 | TGC 15 and CZA 50 |
| *A. baumannii*  I_88_ Ab_4_^*^ | Sputum | 19/25 | P 10, AMP 10, AMC 30, SAM 20, CAZ 30, CFM 5, CRO 30, CTX 30, FEP 30, NOR 10, OFX 5, AK 30, CL 30, ETP 10, MEM 10, CT 10, CZA 50, C/T, and SXT 25 | CFP 75, CIP 5, CN 10, TGC 15, SCF 105, and TZP 110 |
| *A. lwoffii* I_9_ Al_1_^*^ | Chest | 23/25 | P 10, AMP 10, AMC 30, SAM 20, CAZ 30, CFM 5, CFP 75, CRO 30, CTX 30, FEP 30, CIP 5, NOR 10, OFX 5, AK 30,CN 10, CL 30, ETP 10, MEM 10, CZA 50, C/T, SCF 105, SXT 25, and TZP 110 | TGC 15 and CT 10 |
| *E. coli*  I_12_ E_6_ | Urine | 17/25 | P 10, AMP 10, AMC 30, SAM 20, CAZ 30, CFM 5, CFP 75, CRO 30, CTX 30, FEP 30, CIP 5, NOR 10, OFX 5, CN 10, CL 30, C/T, and SXT 25 | AK 30, TGC 15, ETP 10, MEM 10, CT 10, CZA 50, SCF 105, and TZP 110 |
| *E. coli*  I_14_ E_8_ | Wound | 15/25 | P 10, AMP 10, AMC 30, SAM 20, CAZ 30, CFM 5, CFP 75, CRO 30, CTX 30, FEP 30, CIP 5, NOR 10, OFX 5, CN 10, and CL 30 | AK 30, TGC 15, ETP 10, MEM 10, CT 10, CZA 50, C/T, SCF 105, SXT 25, and TZP 110 |
| *E. coli*  I_93_E_34_ | Urine | 22/25 | P 10, AMP 10, AMC 30, SAM 20, CAZ 30, CFM 5, CFP 75, CRO 30, CTX 30, FEP 30, CIP 5, NOR 10, OFX 5, AK 30, CN 10, CL 30, ETP 10, MEM 10, C/T, SCF 105, SXT 25, and TZP 110 | TGC 15, CT 10, and CZA 50 |
| *H. alvei*  I_65_ H_1_ | Blood | 22/25 | P 10, AMP 10, AMC 30, SAM 20, CAZ 30, CFM 5, CFP 75, CRO 30, CTX 30, FEP 30, CIP 5, NOR 10, OFX 5, AK 30, CN 10, CL 30, ETP 10, MEM 10, C/T, SCF 105, SXT 25, and TZP 110 | TGC 15, CT 10, and CZA 50 |
| *K. pneumoniae*  I_33_ Kp_4_ | Liver pus | 23/25 | P 10, AMP 10, AMC 30, SAM 20, CAZ 30, CFM 5, CFP 75, CRO 30, CTX 30, FEP 30, CIP 5, NOR 10, OFX 5, AK 30,CN 10, CL 30, ETP 10, MEM 10, CZA 50, C/T, SCF 105, SXT 25, and TZP 110 | TGC 15 and CT 10 |
| *K. pneumoniae*  I_56_ Kp_6_^*^ | Sputum | 22/25 | P 10, AMP 10, AMC 30, SAM 20, CAZ 30, CFM 5, CFP 75, CRO 30, CTX 30, FEP 30, CIP 5, NOR 10, OFX 5, AK 30, CN 10, CL 30, ETP 10, MEM 10, C/T, SCF 105, SXT 25, and TZP 110 | TGC 15, CT 10, and CZA 50 |
| *K. pneumoniae*  I_89_ Kp_8_^*^ | Sputum | 19/25 | P 10, AMP 10, AMC 30, SAM 20, CAZ 30, CFM 5, CRO 30, CTX 30, FEP 30, NOR 10, OFX 5, AK 30, CN 10, CL 30, ETP 10, MEM 10, CT 10, CZA 50, and SXT 25 | CFP 75, CIP 5, TGC 15, C/T, SCF 105, and TZP 110 |
| *K. ozaenae*  I_2_ Ko_1_^*^ | Sputum | 23/25 | P 10, AMP 10, AMC 30, SAM 20, CAZ 30, CFM 5, CFP 75, CRO 30, CTX 30, FEP 30, CIP 5, NOR 10, OFX 5, AK 30,CN 10, CL 30, ETP 10, MEM 10, CZA 50, C/T, SCF 105, SXT 25, and TZP 110 | TGC 15 and CT 10 |
| *K. ozaenae*  I_80_ Ko_2_ | Urine | 22/25 | P 10, AMP 10, AMC 30, SAM 20, CAZ 30, CFM 5, CFP 75, CRO 30, CTX 30, FEP 30, CIP 5, NOR 10, OFX 5, AK 30, CN 10, CL 30, ETP 10, MEM 10, C/T, SCF 105, SXT 25, and TZP 110 | TGC 15, CT 10, and CZA 50 |
| *K. ozaenae*  I_83_ Ko_3_ | Abscess | 22/25 | P 10, AMP 10, AMC 30, SAM 20, CAZ 30, CFM 5, CFP 75, CRO 30, CTX 30, FEP 30, CIP 5, NOR 10, OFX 5, AK 30, CN 10, CL 30, ETP 10, MEM 10, C/T, SCF 105, SXT 25, and TZP 110 | TGC 15, CT 10, and CZA 50 |
| *K. ozaenae*  I_85_ Ko_4_ | Blood | 22/25 | P 10, AMP 10, AMC 30, SAM 20, CAZ 30, CFM 5, CFP 75, CRO 30, CTX 30, FEP 30, CIP 5, NOR 10, OFX 5, AK 30, CN 10, CL 30, ETP 10, MEM 10, C/T, SCF 105, SXT 25, and TZP 110 | TGC 15, CT 10, and CZA 50 |
| *P. aeruginosa*  I_37_ P_3_^*^ | Sputum | 24/25 | P 10, AMP 10, AMC 30, SAM 20, CAZ 30, CFM 5, CFP 75, CRO 30, CTX 30, FEP 30, CIP 5, NOR 10, OFX 5, AK 30, CN 10, TGC 15, CL 30, ETP 10, MEM 10, CZA 50, C/T, SCF 105, SXT 25, and TZP 110 | CT 10 |
| *P. aeruginosa*  I_100_ P_8_ | Urine | 25/25 | P 10, AMP 10, AMC 30, SAM 20, CAZ 30, CFM 5, CFP 75, CRO 30, CTX 30, FEP 30, CIP 5, NOR 10, OFX 5, AK 30, CN 10, TGC 15, CL 30, ETP 10, CT 10, MEM 10, CZA 50, C/T, SCF 105, SXT 25, and TZP 110 | -- |
| *S. liquefaciens* I_38_ Sel_1_^*^ | Sputum | 23/25 | P 10, AMP 10, AMC 30, SAM 20, CAZ 30, CFM 5, CFP 75, CRO 30, CTX 30, FEP 30, CIP 5, NOR 10, OFX 5, AK 30, CN 10, CL 30, ETP 10, MEM 10, CZA 50, C/T, SCF 105, SXT 25, and TZP 110 | TGC 15 and CT 10 |
| *S. rubidaea* I_48_ Ser_1_^*^ | Chest | 22/25 | P 10, AMP 10, AMC 30, SAM 20, CAZ 30, CFM 5, CFP 75, CRO 30, CTX 30, FEP 30, CIP 5, NOR 10, OFX 5, AK 30, CN 10, CL 30, ETP 10, MEM 10, C/T, SCF 105, SXT 25, and TZP 110 | TGC 15, CT 10, and CZA 50 |
| CON *Staphylococcus* I_84_ St_-5_ | Blood | 25/30 | P 10, AMP 10, AMC 30, SAM 20, CAZ 30, CFM 5, CFP 75, CRO 30, CTX 30, FEP 30, CIP 5, NOR 10, OFX 5, CL 30, ETP 10, CT 10, CZA 50, C/T, SXT 25, TZP 110, DA 2, E 15, LZD 30, OX 1, and TEC 30, | AK 30, CN 10, TGC 15,, MEM 10, andSCF 105 |
| CON *Staphylococcus* I_92_ St_-6_ | Abscess | 18/30 | P 10, AMP 10, AMC 30, CFM 5, CRO 30, FEP 30, CIP 5, NOR 10, OFX 5, CN 10, CL 30, ETP 10, CT 10, MEM 10, SXT 25, DA 2, E 15 and OX 1 | SAM 20,CAZ 30, CFP 75, CTX 30, AK 30, TGC 15, CZA 50, C/T, SCF 105, TZP 110, LZD 30, and TEC 30 |
| *S. aurus*  I_41_St_2_^*^ | Sputum | 23/30 | P 10, AMP 10, AMC 30, SAM 20, CAZ 30, CFM 5, CFP 75, CRO 30, CTX 30, FEP 30, CIP 5, NOR 10, OFX 5, CL 30, ETP 10, CT 10, MEM 10, CZA 50, C/T, SXT 25, TZP 110, DA 2, E 15, and OX 1 | FEP 30, AK 30, CN 10, TGC 15, SCF 105, LZD 30, and TEC 30 |
| *S. aurus*  I_44_ St_3_ | Blood | 26/30 | P 10, AMP 10, AMC 30, SAM 20, CAZ 30, CFM 5, CFP 75, CRO 30, CTX 30, FEP 30, CIP 5, NOR 10, OFX 5, CN 10, CL 30, ETP 10, CT 10, MEM 10, CZA 50, C/T, SCF 105, SXT 25, DA 2, E 15, LZD 30, and OX 1 | AK 30, TGC 15, TZP 110, and TEC 30 |
| *C. albicans*  I_39_ Ca_7_ | Urine | 3/4 | Clotrimazole 10 μg, Fluconazole 25 μg, and Itraconazole 10 μg | Nystatin 100 U |
| *C. albicans*  I_60_ Ca_8_^*^ | Sputum | 3/4 | Clotrimazole 10 μg, Fluconazole 25 μg, and Itraconazole 10 μg | Nystatin 100 U |
| *C. albicans*  I_72_ Ca_12_^*^ | Sputum | 3/4 | Clotrimazole 10 μg, Fluconazole 25 μg, and Itraconazole 10 μg | Nystatin 100 U |
| *C. glabrata*  I_66_ Cg_1_^*^ | Sputum | 3/4 | Clotrimazole 10 μg, Fluconazole 25 μg, and Itraconazole 10 μg | Nystatin 100 U |
| *C. krusei*  I_59_ Ck_1_ | Urine | 3/5 | Clotrimazole 10 μg, Fluconazole 25 μg, and Itraconazole 10 μg | Nystatin 100 U |
| *C. tropicalis*  I_23_ Ct_1_^*^ | Chest | 1/4 | Fluconazole 25 μg | Clotrimazole 10 μg, Itraconazole 10 μg, and Nystatin 100 U |

**P:** Penicillins, **AMP 10**: Ampicillin, **AMC 30:** Amoxicillin-clavulanic acid, **SAM 20:** Sulbactam/ampicillin, CAZ 30: Ceftazidime, **CFM 5:** cefixime, **CFP 75:** cefoperazone, **CRO 30**: Ceftriaxone, **CTX 30**: **Cefotaxime, FEP 30**: Cefepime, **CIP 5:**Ciprofloxacin, **NOR 10:** Norfloxacin, **OFX 5:**ofloxacin, **AK 30:** Amikacin, **CN 10:**Gentamicin, TGC 15: Glycylcycline, **CL 30**: Chloramphenicol, **ETP 10**: Ertapenem, **MEM 10**: Meropenem, **CT 10**: Colistin, **CZA 50**: ceftazidime-avibactam, **C/T:** ceftolozane/tazobactam, **SCF 105**: Cefoperazone/Sulbactam, **SXT 25**: Sulphamethoxazole/trimethoprim, **TZP 110:** piperacillin/tazobactam, **DA 2**: Clindamycin, **E 15**: Erythromycin, **LZD 30**: linezolid,**OX 1**: Oxacillin, and **TEC 30:** teicoplanin

**
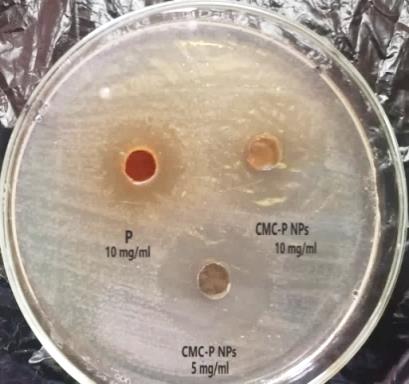

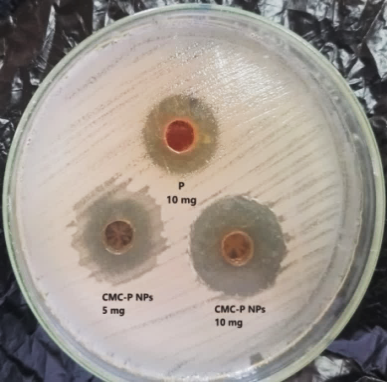

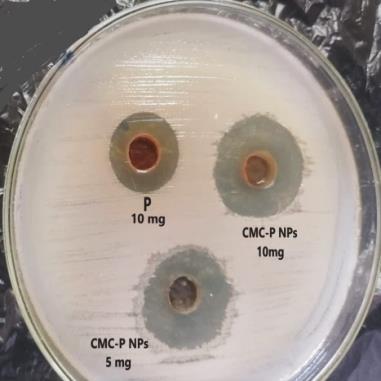
**

***K. pneumonia***

***K. pneumonia***

***A. baumannii***

***K. ozaenae***

***A. baumannii***

**
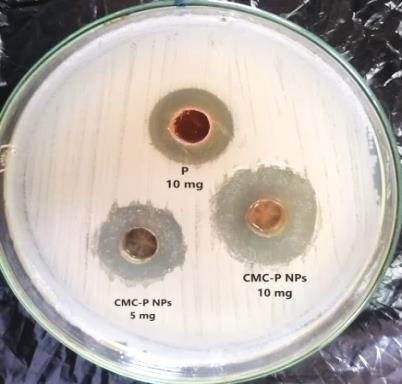

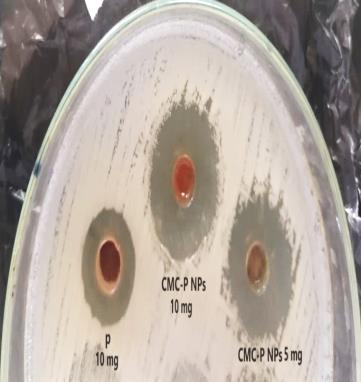

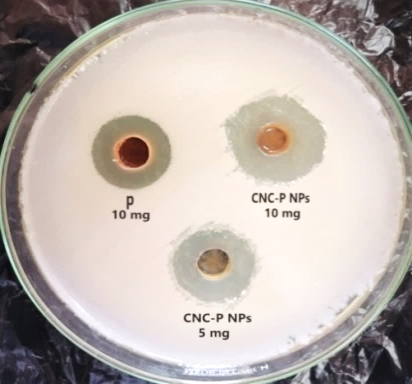
**

***K. ozaenae***

**
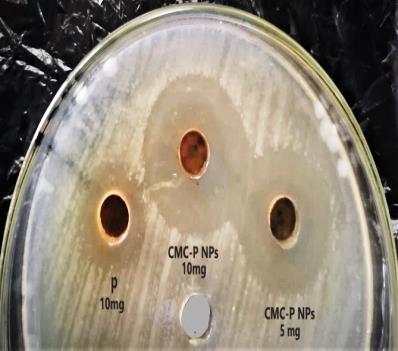

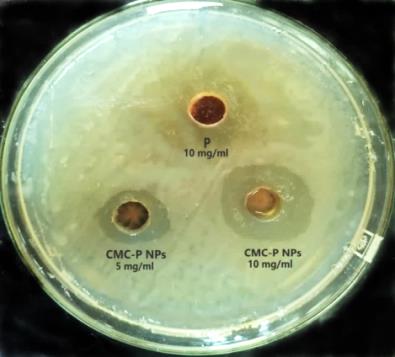

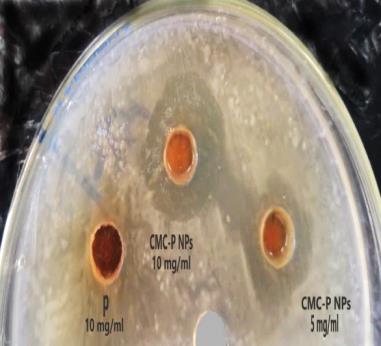
**

***S. liquefaciens***

***P. aurginosa***

***H. alvei***

**
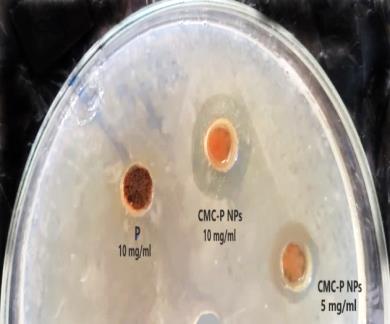

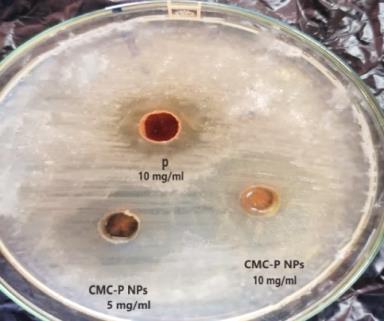

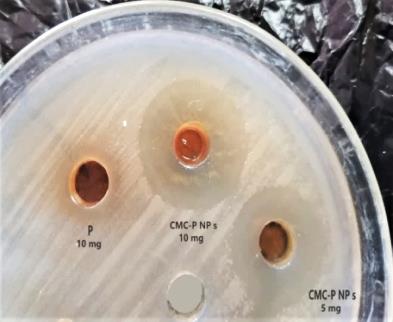
**

***S. rubidaea***

**CON *Staphylococcus* spp.**

***S. aureus***

**
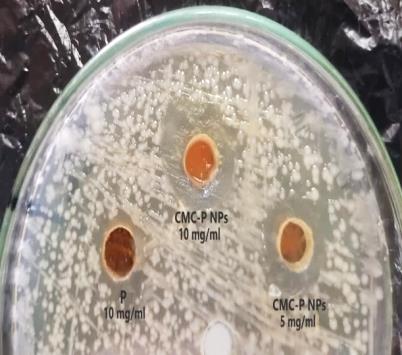

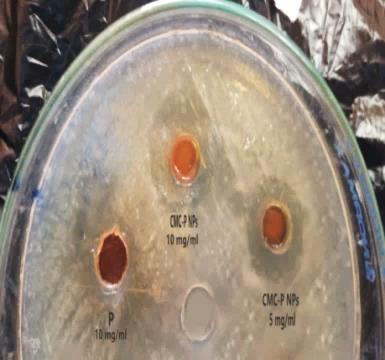

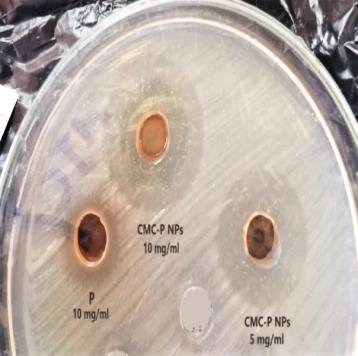
**

***C. tropicalis***

***C. krusei***

***C. albicans***

**Supplementary Plate (1):** Plate’s photos reveals antimicrobial activity of propolis (P) (10 mg/ml) and CMC-P NPs (10 and 5 mg/ml) on some MDR Gram-negative, Gram-positive bacteria, and yeast isolates under study.


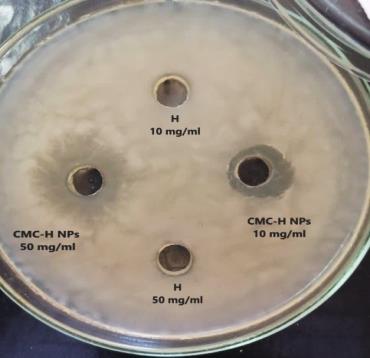

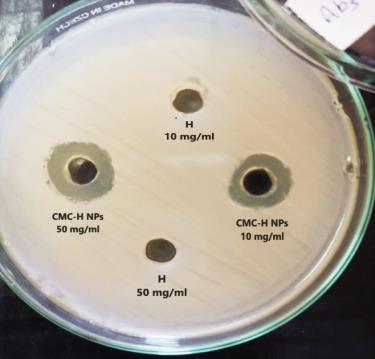

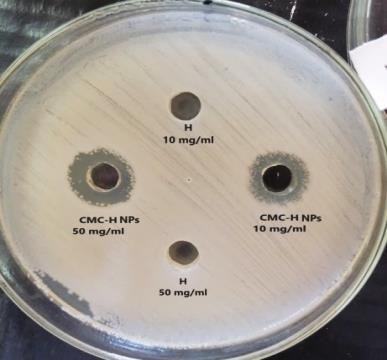


***A. lwoffii***

***A. baumannii***

***K. ozaenae***


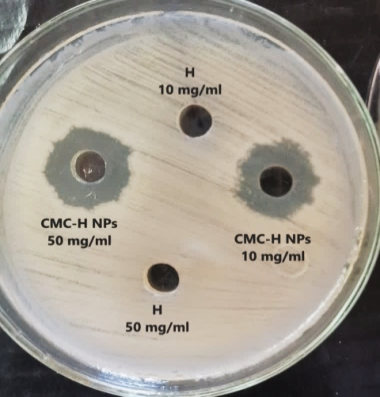
 **
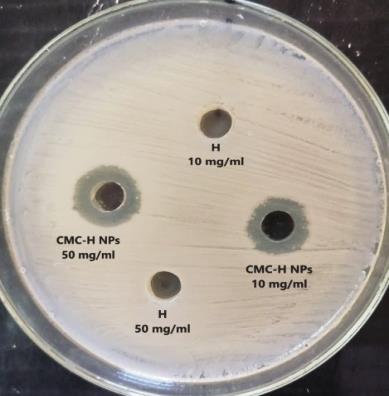
**
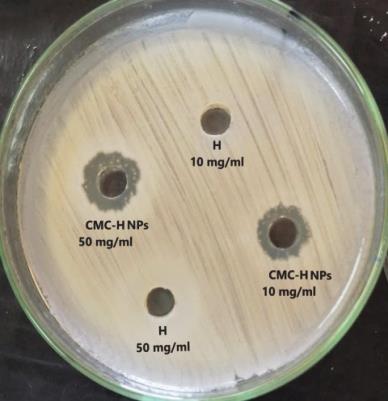


***K. ozaenae***

***K. pneumonia***

***K. pneumonia***

**
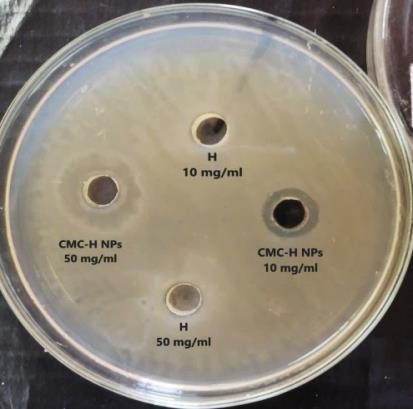

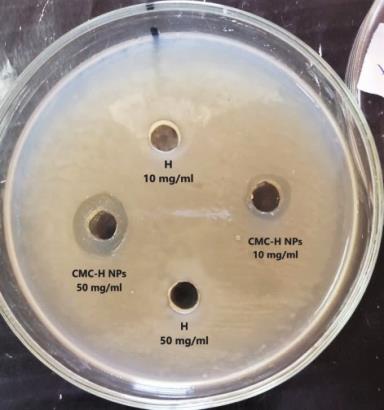

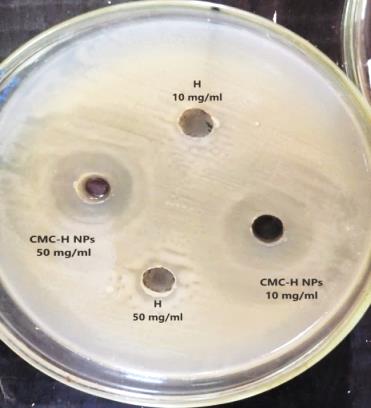
**

***S. liquefaciens***

***H. alvei***

***E. coli***

**
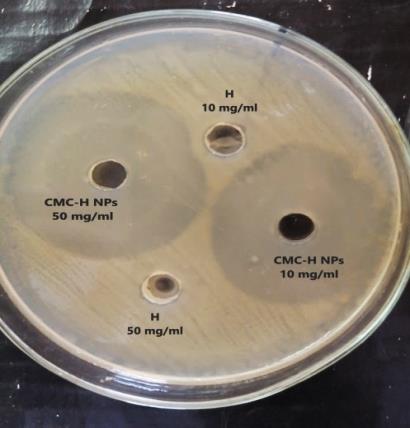

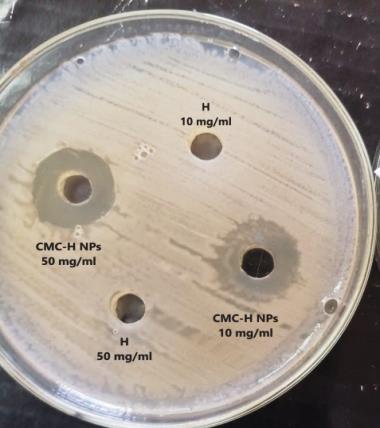

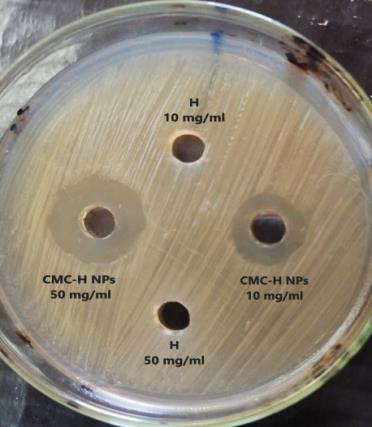
**

**CON *Staphylococcus* spp.**

***S. rubidaea***

***S. aureus***

**
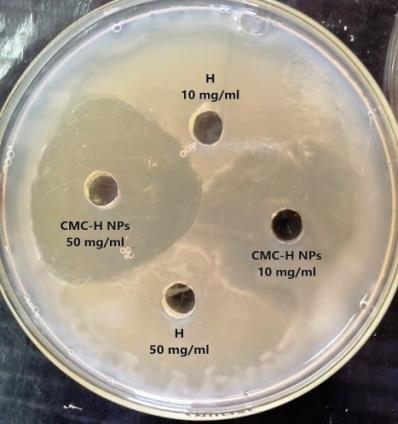

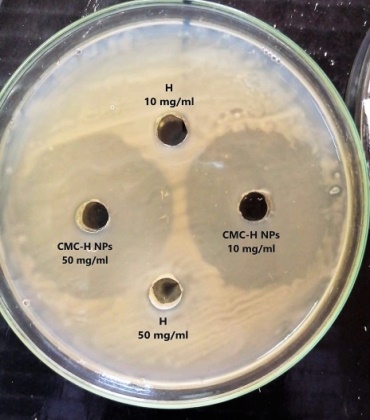

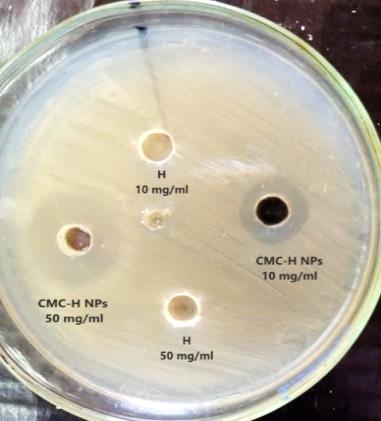
**

***C. tropicalis***

***C. glabrata***

***C. albicans***

**Supplementary Plate (2):** Plate’s photos reveals antimicrobial activity of Egyptian honey (H) (50 and 10 mg/ml) and CMC-H NPs (50 and 10 mg/ml) on some MDR Gram-negative, Gram-positive bacteria, and yeast isolates under study

***E. coli***

***A. baumannii***

**
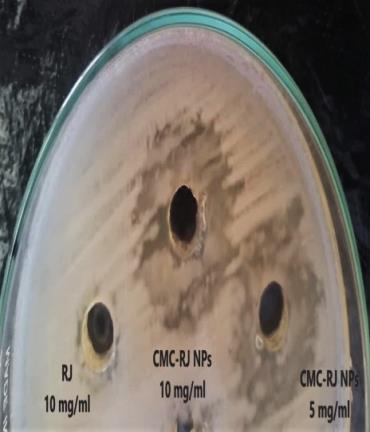

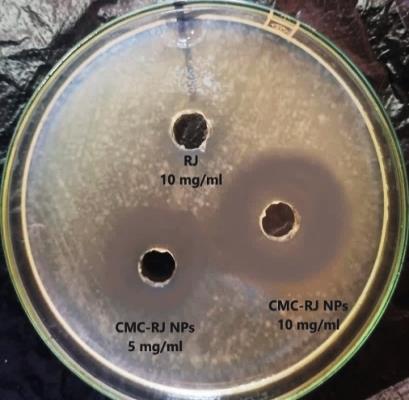

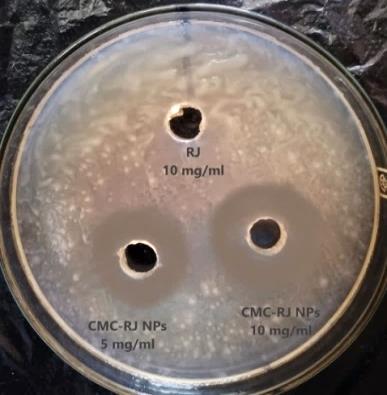
**

***E. coli***

**
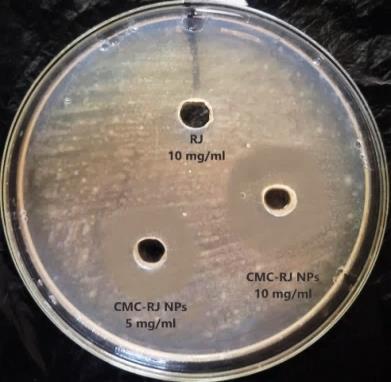

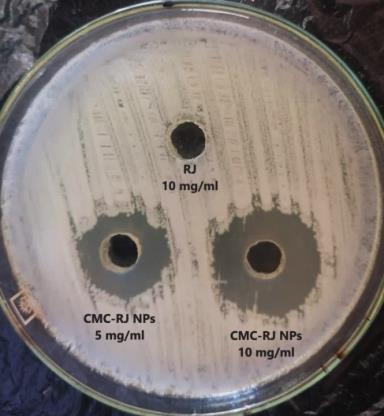

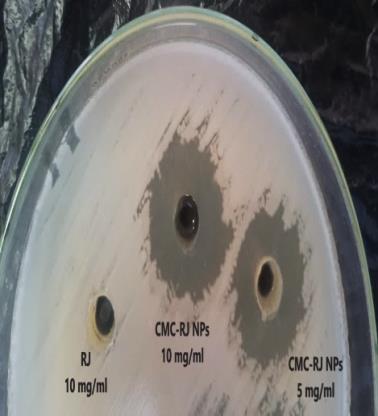
**

***K. ozaenae***

***H. alvei***

***K. ozaenae***

**
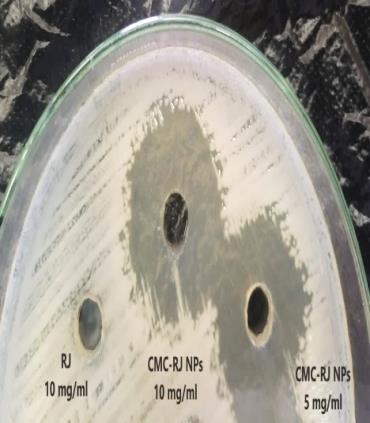

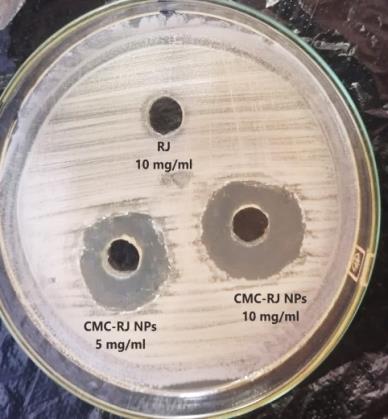

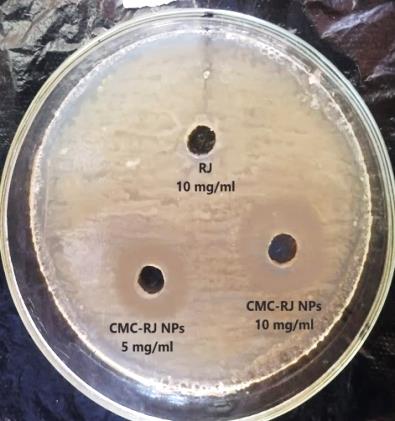
**

***S. aureus***

***K. pneumonia***

***K. pneumonia***

**Supplementary Plate (3):** Plate’s photos reveals antimicrobial activity of royal jelly (RJ) (10 mg/ml) and CMC-RJ NPs (10 and 5 mg/ml) on some MDR Gram-negative, Gram-positive bacteria, and yeast isolates under study.
